# Diversity and Functionality of Bacteria Associated with Different Tissues of Spider *Heteropoda venatoria* Revealed through Integration of High-Throughput Sequencing and Culturomics Approaches

**DOI:** 10.1007/s00248-024-02383-2

**Published:** 2024-05-04

**Authors:** Likun Zhao, Shanfeng Zhang, Ruoyi Xiao, Chao Zhang, Zhitang Lyu, Feng Zhang

**Affiliations:** 1 College of Life Sciences, Institute of Life Sciences and Green Development, Hebei University, Baoding, 071002 People’s Republic of China; 2 The Key Laboratory of Microbial Diversity Research and Application of Hebei Province, Baoding, 071002 People’s Republic of China; 3 The Key Laboratory of Zoological Systematics and Application of Hebei Province, Baoding, 071002 People’s Republic of China

**Keywords:** Spider-associated bacteria, High-throughput sequencing, Culturomics, Bioactivity

## Abstract

Spiders host a diverse range of bacteria in their guts and other tissues, which have been found to play a significant role in their fitness. This study aimed to investigate the community diversity and functional characteristics of spider-associated bacteria in four tissues of *Heteropoda venatoria* using HTS of the 16S rRNA gene and culturomics technologies, as well as the functional verification of the isolated strains. The results of HTS showed that the spider-associated bacteria in different tissues belonged to 34 phyla, 72 classes, 170 orders, 277 families, and 458 genera. *Bacillus* was found to be the most abundant bacteria in the venom gland, silk gland, and ovary, while *Stenotrophomonas*, *Acinetobacter*, and *Sphingomonas* were dominant in the gut microbiota*.* Based on the amplicon sequencing results, 21 distinct cultivation conditions were developed using culturomics to isolate bacteria from the ovary, gut, venom gland, and silk gland. A total of 119 bacterial strains, representing 4 phyla and 25 genera, with *Bacillus* and *Serratia* as the dominant genera, were isolated. Five strains exhibited high efficiency in degrading pesticides in the *in vitro* experiments. Out of the 119 isolates, 28 exhibited antibacterial activity against at least one of the tested bacterial strains, including the pathogenic bacteria *Staphylococcus aureus*, *Acinetobacter baumanii*, and *Enterococcus faecalis*. The study also identified three strains, GL312, PL211, and PL316, which exhibited significant cytotoxicity against MGC-803. The crude extract from the fermentation broth of strain PL316 was found to effectively induce apoptosis in MGC-803 cells. Overall, this study offers a comprehensive understanding of the bacterial community structure associated with *H. venatoria*. It also provides valuable insights into discovering novel antitumor natural products for gastric cancer and xenobiotic-degrading bacteria of spiders.

**Supplementary Information:**

The online version contains supplementary material available at 10.1007/s00248-024-02383-2.

## Introduction

The gut microbiome is known to play a crucial role in maintaining the overall health of its host. Spiders, as one of the largest groups of arthropods besides insects, are home to 50,708 known species worldwide [1], with a diverse range of symbiotic bacteria present in their bodies [2, 3]. The development of high-throughput sequencing technology (HTS) has sparked increasing interest in the bacterial community within spiders [4–8]. However, HTS can sometimes fail to detect low-abundance microorganisms and is subject to limitations such as cross-study bias due to differences in DNA extraction methods and target regions, as well as a lack of sensitivity to distinguish certain bacterial genera [9, 10]. Moreover, isolation is crucial for screening the production of secondary metabolites for drug discovery. Therefore, even in the “omics” era, pure culture remains an essential and reliable method for fully characterizing a given microorganism and obtaining definitive information about its phylogenetic, metabolic, and ecological relevance, as well as its biotechnological potential [11].

Culturomics, which utilizes diverse culture conditions and innovative protocols, has facilitated the isolation of a wider variety of microorganisms from different habitats, thereby maximizing the ability to fulfill the requirements for microbial isolation [12]. In addition, the bacterial strains obtained through culturomics are suitable for studying bacterial functions at the strain level. They can play a key role in exploring the relationship between microbial communities and hosts [12]. Insects have been extensively studied for their antimicrobial properties [13], as well as their antimicrobial, anticancer, and antiparasitic activity [14, 15]. Nazipi et al. also investigated the antimicrobial potential of bacteria isolated from spider nests [16]. However, the diversity and function of spider-associated bacteria are not well understood. We hypothesize that there is a diverse range of spider-associated bacteria and these bacteria are linked to their venom function and environmental resistance. Therefore, we employ both HTS and culturomics methods to analyze the relative abundance and diversity of both culturable and non-culturable bacteria in various tissues (gut, ovary, venom gland, and silk gland) of *H. venatoria*. This approach offers new insights into the community structure of spider-associated bacteria and aims to elucidate the function of cultivable bacterial strains of *H. venatoria*.

For the first time, the bioactivity potential of gut-associated bacteria isolated from spiders was reported, including their abilities to degrade pesticides, exert antibacterial effects, and inhibit tumor cell growth.

## Materials and Methods

### Sample Collection and Dissection

**Fig. 1:**
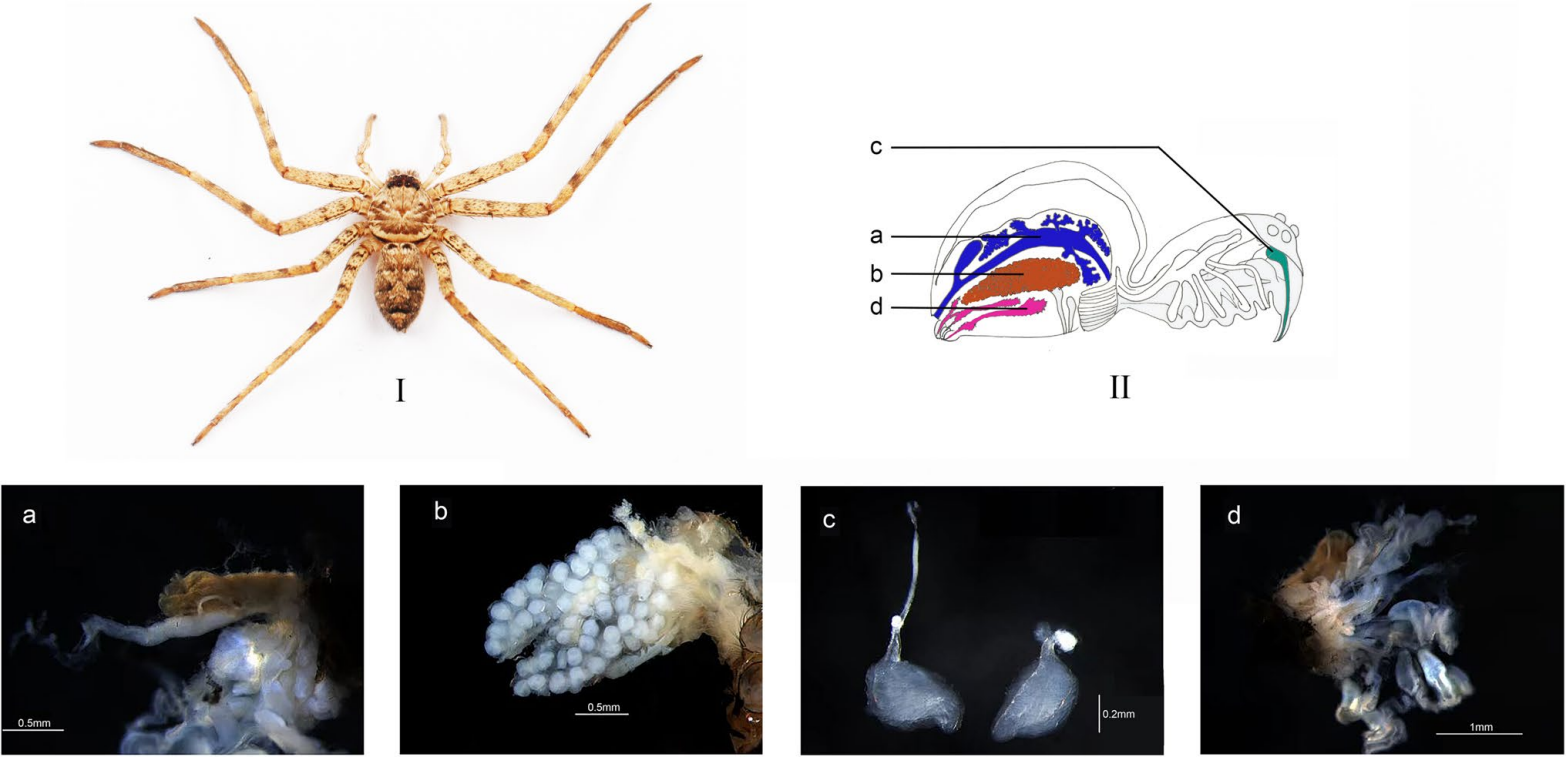
*H. venatoria* and four anatomical tissues*.* (I) Photograph of *H. venatoria*, dorsal view. (II) Schematic spider in anatomy, lateral view. **a**–**d** Four tissues of *H. venatoria*. **a** Gut tissue. **b** Ovary tissue. **c** Venom gland. **d** Silk gland

Around 300 adult spiders were collected from Guangchang County [40°19′N, 116°6′E], Fuzhou City, Jiangxi Province. After not eating for 5 days [2], the specimens were rinsed with distilled water and then subjected to the following washes: 1 min in 100% ethanol; 3 min in 2.5% NaOCl; 1 min in 100% ethanol; 30 s in distilled water, repeated three times. Subsequently, various tissues, such as the gut, ovary, venom gland, and silk gland, were dissected and separated using sterile forceps under aseptic conditions (Fig. 1). Each tissue type was randomly divided into three biological replicates. The tissues were named G1–G3 for the spider gut, V1–V3 for the venom gland, S1–S3 for the silk gland, and O1–O3 for the ovary tissue. Finally, the tissues were stored at − −80 °C until they were used to extract metagenomics DNA for HTS.

The same processing procedure was used to isolate spider-associated bacteria. Approximately five spider tissues (raised under equal conditions) were mixed for single isolation. To verify surface sterility, a sample of the final rinsing water was cultured on an LB medium plate at 37 °C for 48 h.

### High-Throughput Sequencing and Bioinformatic Analysis

The DNA was extracted using the CTAB method, and the V3-V4 region of the 16S rRNA gene was amplified using universal primers: 338F (5′-ACTCCTACGGGAGGCAGCAG-3′) and 806R (5′-GGACTACHVGGGTWTCTAAT-3′) [17]. The amplicons were sequenced using the IonS5™ XL platform, and gene library construction, sequencing, and data analysis were performed by Novogene Bioinformatics (Tianjin, China). Primer sequences were trimmed from merged reads with lengths shorter than 150 bp and were discarded. Filtered and merged sequences were clustered into operational taxonomic units (OTUs) using a threshold of 97% sequence identity threshold after detecting and removing chimeric sequences. Using Qiime2 (v 2020.2) software, Amplicon Sequence Variants (ASVs) clustering was performed on the sequences using the DADA2 algorithm, and chimeras were removed. Based on the Silva 16SrRNA database (v138), the RDP classifier Bayesian algorithm was used to annotate the ASV representative sequences for taxonomic classification (confidence threshold 0.8), obtaining taxonomic annotation results. Use the Kruskal-Wallis rank test to evaluate alpha diversity of microbial communities and correct for multiple testing by adjusting all *P*-values using the False Discovery Rate (FDR) method. Using the “vegan” package (version 2.4.3) in R statistical software, NMDS (non-metric multidimensional scaling) was used to reduce the dimensionality of community structure changes. The similarity analysis software ANOSIM (analysis of similarity) was used to test the differences in bacterial community structure between different organizations, and PERMANOVA (permanent differentiation of variance) was used to evaluate the significance of microbial community structure differences between different organizations. The bipartite network was created to highlight the common genera among samples, employing the div_network function from the gg ClusterNet package in R version 4.2.0.1 [18, 19].

The raw sequencing data for this study can be found on the National Center for Biotechnology Information’s Sequence Read Archive (SRA) website with the accession numbers SAMN27968563 to SAMN27968574.

### Culturomics-Based Bacteria Isolation and Identification

After analyzing the HTS results, we conducted a culturomics study based on a relative abundance table at the genus level. Of the 14 genera with an average abundance above 0.05%, 13 were aerobic or facultative anaerobic bacteria, and only *Parabacteroides* is an obligate anaerobe (SI Table 1). Due to the challenges associated with developing and applying microbial natural products, such as critical culture conditions and difficulty in genetic manipulation, we have chosen to exclude the obligate genus *Parabacteroides* from further analysis.

The predicted media is based on information from the KOMODO (Known Media Database) media recommendation system, as well as the website of the Leibniz Institute DSMZ (http://www.dsmz.de) and JCM (Japan Collection of Microorganisms, https://jcm.brc.riken.jp/en/) [20, 21]. In total, seven predicted media, including LB (DSMZ medium No.381), MRS (DSMZ medium No.11), TSA (DSMZ medium No.535), R AGAR (JCM medium No. 26), BHI (DSMZ medium No.215), C/10 Medium (DSMZ medium NO. 49), and CASO AGAR (DSMZ medium No.220), were subsequently used. The media LB, MRS, BHI, and CASO were provided by Sigma-Aldrich in Shanghai, China, while the others were prepared according to the medium formulation of DSMZ or JCM. The chemicals used were purchased from Solarbio Science & Technology in Beijing, China.

To isolate oligotrophic bacteria, previous studies have shown that reducing nutrient concentrations in the media and extending the incubation time can be effective [22, 23]. To achieve optimal results, dilution media of 1/5 and 1/10 nutrient concentrations were used in addition to the original concentration, resulting in a total of 21 culture conditions for each bacteria isolation.

The similar processing procedure was used to isolate spider-associated bacteria, where approximately five spider tissues were mixed for a single isolation. Each sample was homogenized using a sterile grinding pestle (Sigma-Aldrich, MO, USA), and then the tissue homogenate was diluted 10 ×  and 100 ×  with sterile water. Twenty microliters of both undiluted and diluted homogenates were transferred to separate culture media plates and then incubated for 24 to 120 h until the colonies were observed. The colonies were purified using the streak plate method to obtain pure cultures. The entire DNA of each isolate was extracted using a bacterial genomic DNA extraction kit (Tiangen Biotech, Beijing, China) following the manufacturer’s instructions. Amplification of the 16S rRNA gene sequence was performed using the primers 27F/1492R (27F: 5′-AGA GTT TGA TCM TGG CTC AG-3′ and 1492R: 5′-TAC GGY TAC CTT GTT ACG ACT T-3′) [17] through polymerase chain reaction (PCR). The reaction mixture consisted of 1 µL of template DNA, 12.5 µL of 2 × PCR Master Mix Solution (Tiangen Biotech, Beijing, China), and 1 µL each of the 0.1 µM primers. Ultrapure water was added to reach a total volume of 25 µL [14]. The PCR program consisted of the following steps: (1) 95 °C for 5 min, (2) 94 °C for 45 s, (3) 55 °C for 45 s, (4) 72 °C for 1 min (repeated 30 times), and (5) 72 °C for 10 min. The PCR product was sequenced directly by Sangon Biotech Co., Ltd. (Shanghai, China).

The taxonomical identification of bacterial isolates was conducted using EzBioCloud (http://eztaxon-e.ezbiocloud.net). Sequences with the highest identity score were chosen, and the multiple sequence alignment was performed using the Clustal X software [24]. The neighbor-joining phylogenetic tree was constructed using MEGA X [25]. The datasets were created, annotated, and used to build the interactive tree in Interactive Tree of Life (iTOL). The determined 16S rRNA gene sequences of each strain were submitted to GenBank, and the accession numbers are listed in SI Table 4.

### Pesticide Degradation Profile Determination

Biodegradation reactions were conducted in 500 mL Erlenmeyer flasks containing 50 mL of liquid culture medium. To determine the pesticide degradation profile, isolates were inoculated in mineral salt medium (MSM: KH_2_PO_4_, 0.9 g, Na_2_HPO_4_⋅12H_2_O, 6.5 g, MgSO_4_⋅7H_2_O, 0.2 g, sodium citrate, 3 g, pH 7.0) with 100 mg/L of bifenthrin or atrazine and incubated for 7 days at 30 °C with constant shaking at 180 rpm [26]. The isolates that were able to grow were then re-inoculated in MSM with 200 mg/L of pesticides, and the final concentration was increased to 500 mg/L [27]. The degradation rates of the pesticide were determined for the isolates that tolerated 500 mg/L of pesticides. The experiment was conducted in flasks containing 100 mg/L of pesticides and cultivated for 12 days at 30 °C. The substrates were extracted with an equal volume of chloroform, and a spectrophotometric-chemometric (Spec-Chem) approach was applied to analyze the residual bifenthrin [28] and atrazine every 24 h [29]. Atrazine and bifenthrin (with a purity of 97%) were purchased from Aladdin (https://www.aladdin-e.com/).

### Antibacterial Activity of the Bacterial Isolates

The modified agar well diffusion method was employed to assess the antibacterial activity of bacterial isolates from spiders against *Staphylococcus aureus*, *Enterococcus faecalis*, and *Acinetobacter baumanii* [30]. Bacterial suspensions were prepared from indicator strains grown overnight. The overnight cultures were diluted 100 times in sterile saline water, added to molten nutrient broth agar medium, and then poured into petri dishes. The isolates associated with spider were agitated at 250 rpm for 24 h in LB media. After centrifugation, 200 µL of the supernatant was added to the Oxford cup on the plate in triplicate for each strain. The plates were then incubated at 30 °C and examined for inhibition zones after 24 and 48 h [31]. The diameter of the inhibition zone was measured to evaluate antimicrobial activity [32], with gentamicin used as a positive control. Each inhibition assay was repeated three times, and the size of the inhibition zone was estimated by measurement.

### Anti-gastric Cancer Cell Activity of the Bacteria Isolates

To determine the cytotoxic activity against cancer cells, the bacterial isolates were fermented, and crude extracts were obtained. The isolates were incubated in LB broth for 24 h at 30 °C on a rotary shaker at 150 rpm with 700 mL of medium in a 3 L Erlenmeyer flask. A total of 15 L fermentation broth was filtered and then extracted with ethyl acetate (EtOAc) of each antimicrobial active strain. The EtOAc phase was concentrated under reduced pressure using a rotary film evaporator at 45 °C to obtain the crude extract. The crude extracts were dissolved in dimethyl sulfoxide (DMSO), and the concentration was adjusted to 40, 20, 10, 5, and 2.5 µg/mL for use.

The cytotoxic effects of the crude extract from the fermentation broth were tested using the 3-[4,5-Dimethylthiazol-2-yl] -2,5-diphenyl tetrazolium bromide (MTT) assay [33]. The human gastric cancer cell line MGC-803 was inoculated into 96-well plates and divided into the control group and the experimental group. The crude extracts were added to the well during the logarithmic growth phase and then cultured for 24 h. Five replicates were prepared for each treatment. At the end of the cultivation, MTT and DMSO were added to the cell cultures, and the OD values for each treatment group at 570 nm were measured using the MTT assay [34]. Finally, the survival ratio of the cells and IC_50_ were calculated based on the detection results.

## Results

### Culture-Independent Analyses of Spider-Associated Bacterial Communities in *H. venatoria* Tissues

**Fig. 2:**
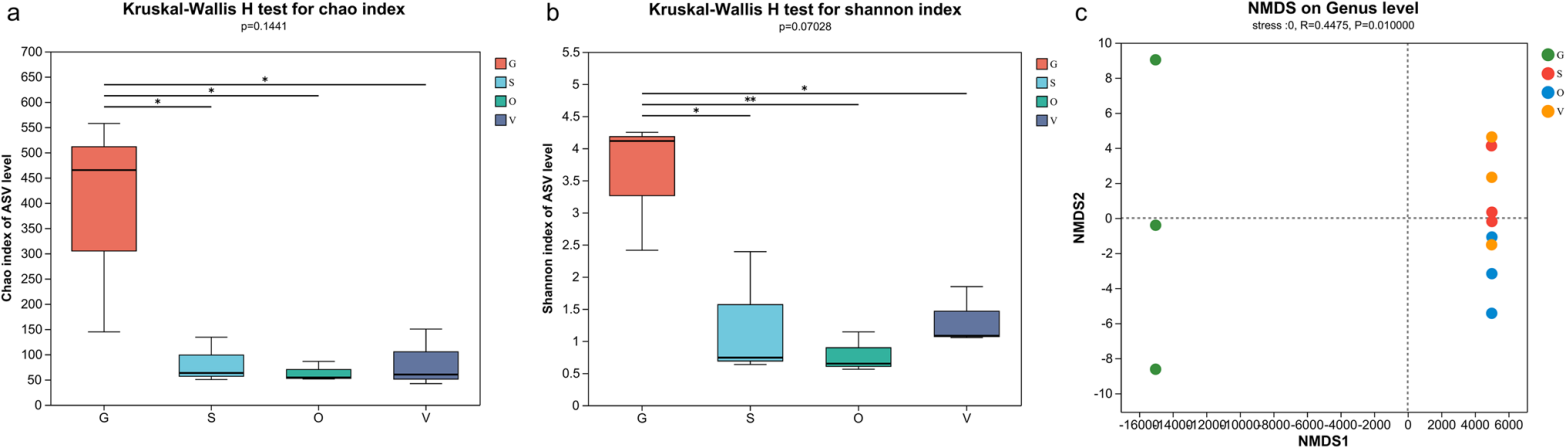
Alpha-diversity and beta-diversity of the microbial community of the different tissues. **a** Chao index. **b** Shannon index. **c** The NMDS analysis at ASV cluster level

Culture-independent analyses were performed to investigate the symbiotic bacterial communities. Sequencing of the 16S rRNA gene amplicons yielded an average of 81,121 reads per sample, and the reads for each sample are listed in SI Table 2. These sequences were subsequently assigned to 1392 ASVs. These ASVs represented a diverse range of taxonomic levels, including 34 bacterial phyla, 73 classes, 165 orders, 260 families, and 497 species across 427 genera. In the diversity index, a higher Chao index value indicates greater species richness, while a higher Shannon index value signifies increased community diversity. The alpha-diversity demonstrated that the gut exhibited the highest community richness and diversity (Fig. 2a, b). PERMANOVA (*R*^2^ =  0.8190, *P* =  0.016) and ANOSIM (*P* =  0.0080) were employed to analyze and elucidate differences between groups. The beta diversity findings suggest that the bacterial communities in the three tissues, silk gland, venom, and ovary, except for the gut, are relatively more similar to each other, showing significant differences from the gut microbiota (Fig. 2c).

**Fig. 3:**
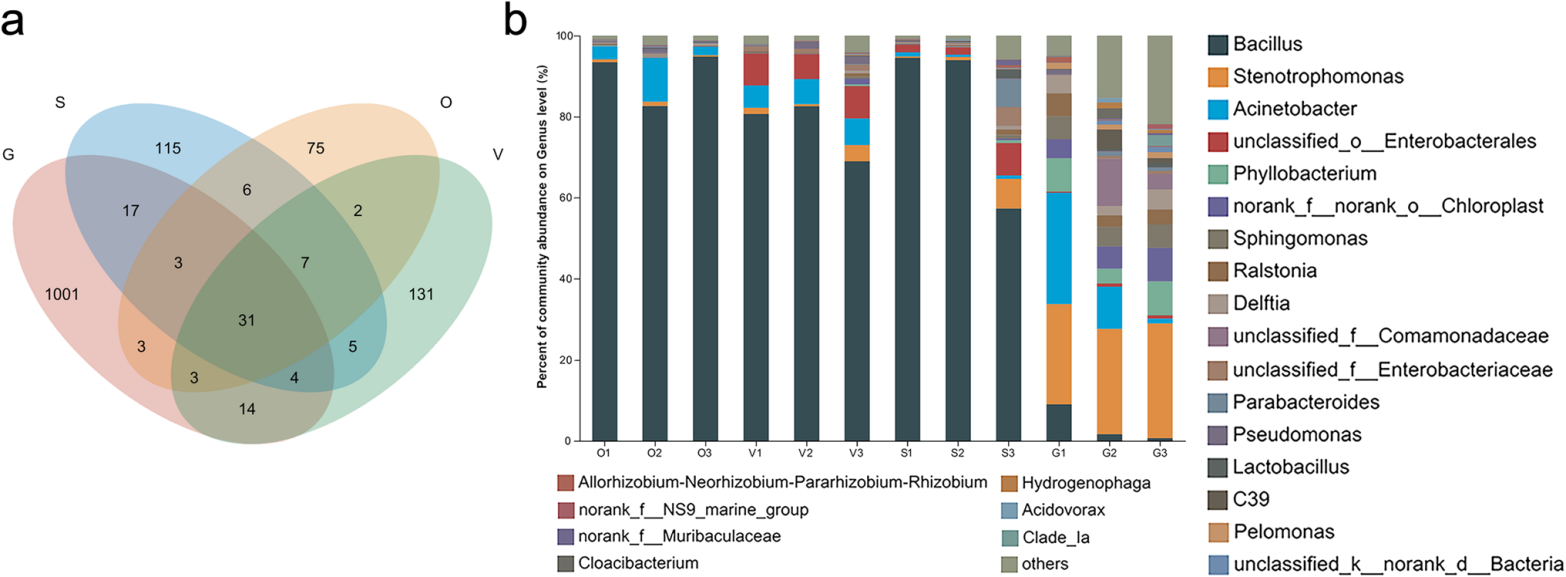
Bacterial community variation among different tissues in *H. venatoria*. **a** Venn diagram showing ASVs classification in the ovary, venom gland, silk gland, and gut. **b** Relative abundance of bacterial communities among the ovary, venom gland, silk gland, and gut in *H. venatoria* at the genus level

The gut had the highest number of ASVs at 1076, with 1001 of them being unique, accounting for 56% of the gut-associated OTUs (Fig. 3a). The genus *Bacillus* was predominant in the venom gland, silk gland, and ovary, while *Stenotrophomonas*, *Acinetobacter*, and *Sphingomonas* were prevalent in the gut (Fig. 3b).

**Fig. 4:**
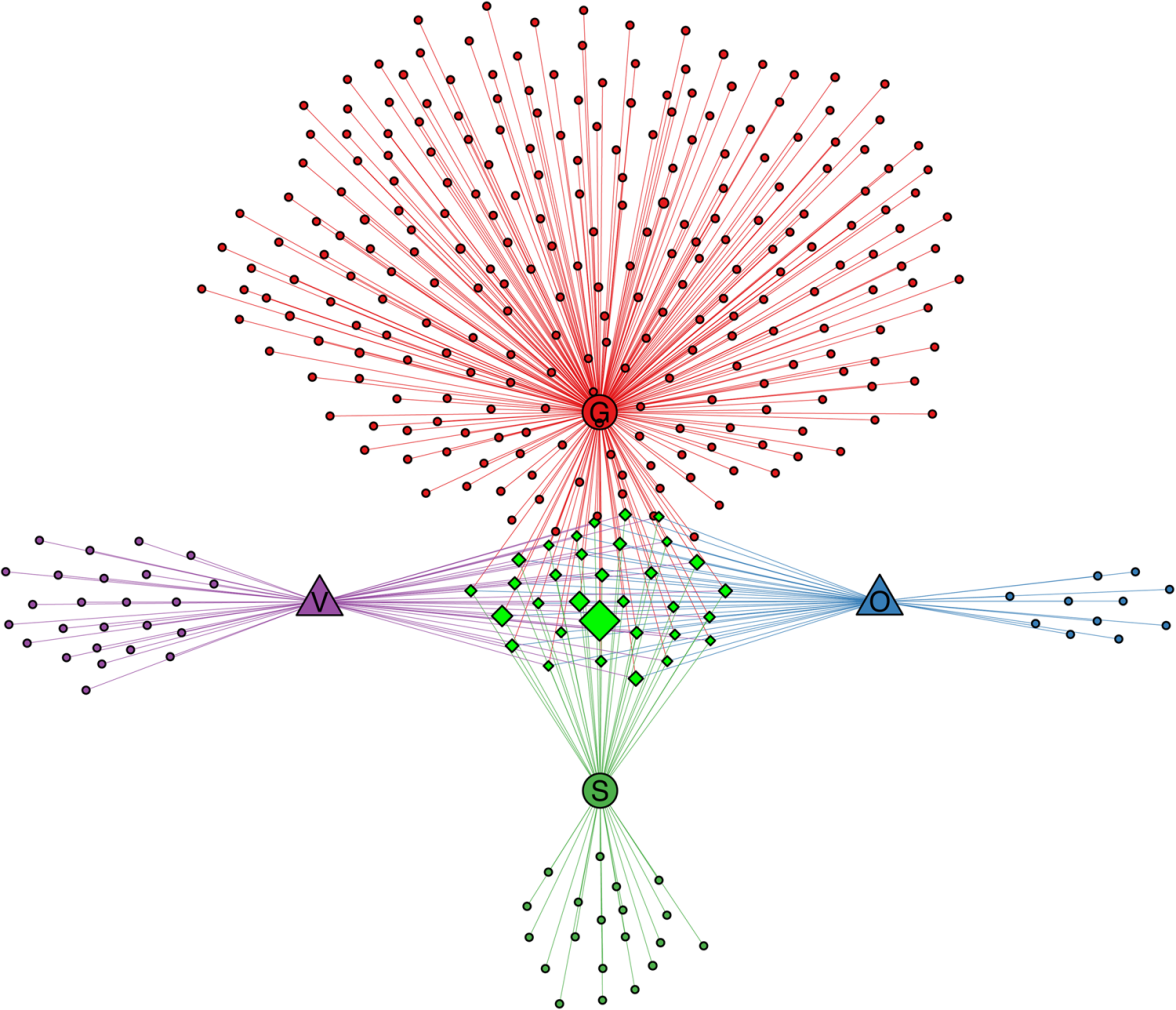
Bipartite network analysis among tissue-associated bacteria. Each node represents one genera and the line colors denote the different tissues. The size of nodes roughly represents the average abundance of the genus. The nodes that are not separated in the middle are shared among all four groups, whereas the remaining nodes are unique to each individual group. G, gut; O, ovary; V, venom gland; S, silk gland

The bipartite network analysis reveals a total of 32 common genera across the four tissues. The gut demonstrates the highest number of unique genera, totaling 250, consistently displaying diversity. Additionally, venom glands exhibit the highest count of endemic genera among the other three tissues, with 25 unique genera. Furthermore, venomous glands stand out with the highest count of exclusive genera among the other three tissues, featuring 25 unique genera. Additionally, both the silk glands and ovaries host their own set of tissue-specific genera, comprising 20 and 11 genera, respectively (Fig. 4, SI Table 3).

### Culturable Bacterial Diversity of the *H. venatoria* Tissues

**Fig. 5:**
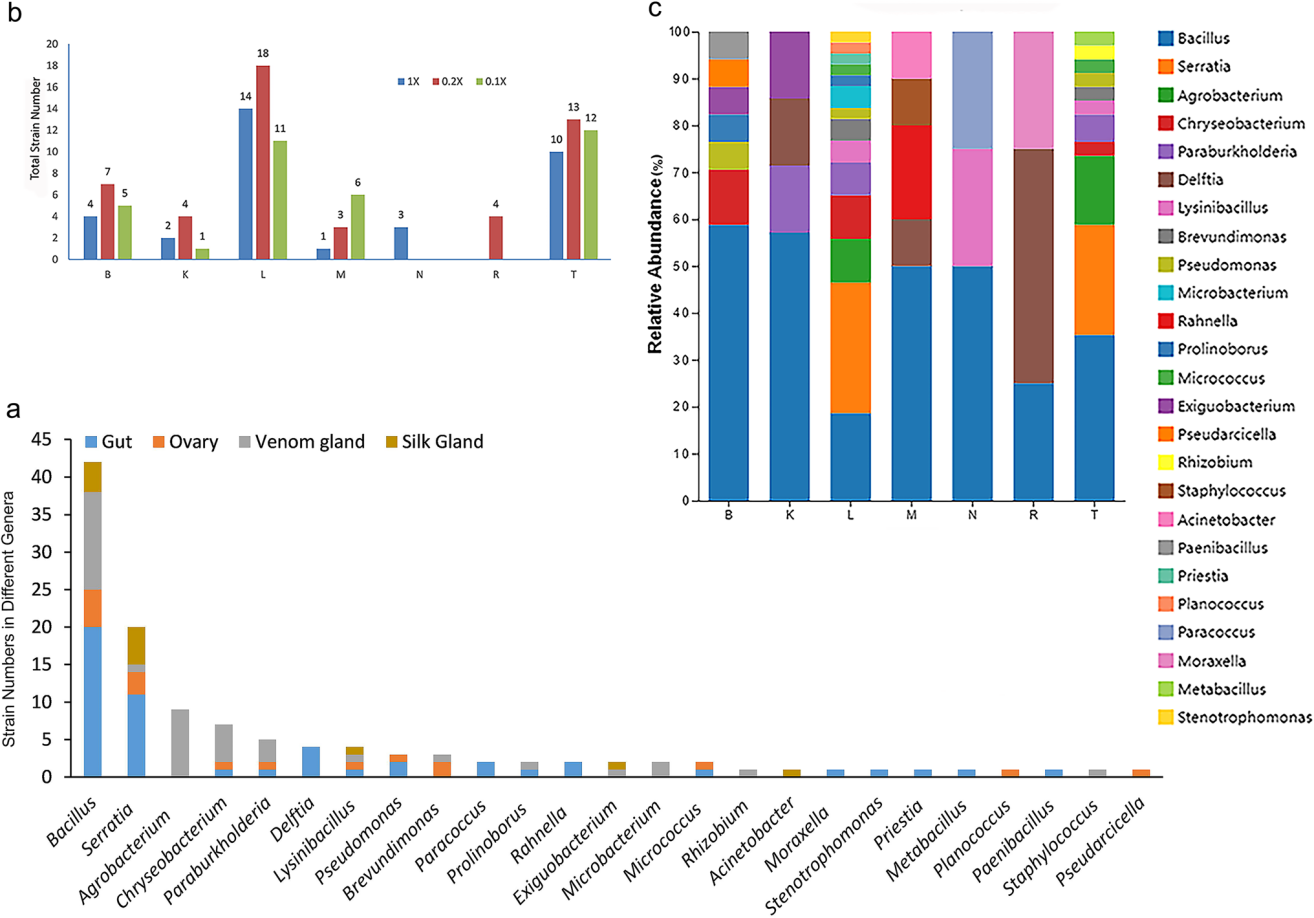
Analysis of the bacteria isolated from the spiders. **a** Strain numbers of each genus in different tissues. **b** The number of bacteria isolated from different culture conditions. **c** Diversity and abundance of the cultivable bacteria isolated from the seven media. B, BHI; K, CASO AGAR; L, LB; M, MRS; N, C/10 MEDIUM; R, R AGAR; T, TSA

A total of 215 clones were initially analyzed from the isolation plates, and 119 representative isolates were selected for phylogenetic analysis based on 16S rRNA gene sequences (SI Table 4). To assess the diversity of cultivable bacteria in the four tissues under investigation, the number of bacteria categorized by genus was tabulated (SI Table 5). In Fig. 5a, it was revealed that the bacterial isolates belonged to 25 genera across four phyla: Proteobacteria (54 strains), Firmicutes (53 strains), Bacteroidota (8 strains), and Actinobacteriota (4 strains).

In this study, all isolates were obtained from 17 out of the 21 culture conditions used for isolation. No bacteria grew on the 1/5 C/10, 1/10 C/10 medium, R agar, and 0.1 ×  R agar. LB and TSA media yielded a greater number of strains, with the highest number of strains obtained at the 0.2 ×  media concentration (Fig. 5b). Comparisons at the genus level of the cultured bacteria obtained using the seven different media revealed that LB and TSA media supported the growth of a more diverse range of genera. The genus *Bacillus* exhibited higher relative abundance and diversity on all five media, except for LB and R agar (Fig. 5c).

According to the findings, six out of the originally targeted 13 aerobic bacterial genera with an abundance over 0.05%, *Acinetobacter*, *Bacillus*, *Chryseobacterium*, *Delftia*, *Pseudomonas*, and *Stenotrophomonas*, were isolated and identified. Additionally, nine genera with lower abundance (less than 0.05%), including *Paraburkholderia*, *Brevundimonas*, *Paracoccus*, *Rhizobium*, *Paenibacillus*, *Staphylococcus*, *Pseudarcicella*, *Microbacterium*, and *Micrococcus*, were also identified. However, the remaining ten genera were not detected through HTS. Almost half (51 strains, 42.9%) of the isolates originated from the gut, while the silk gland yielded the fewest isolates (12 strains, 10.1%). In general, *Bacillus* and *Serratia* were the most prevalent genera found. *Bacillus* was more frequently isolated from the gut, venom gland, and ovary, while *Serratia* was more commonly isolated from the silk gland (SI Table 4).

### Comparison of Results from HTS and Culturomics

**Fig. 6:**
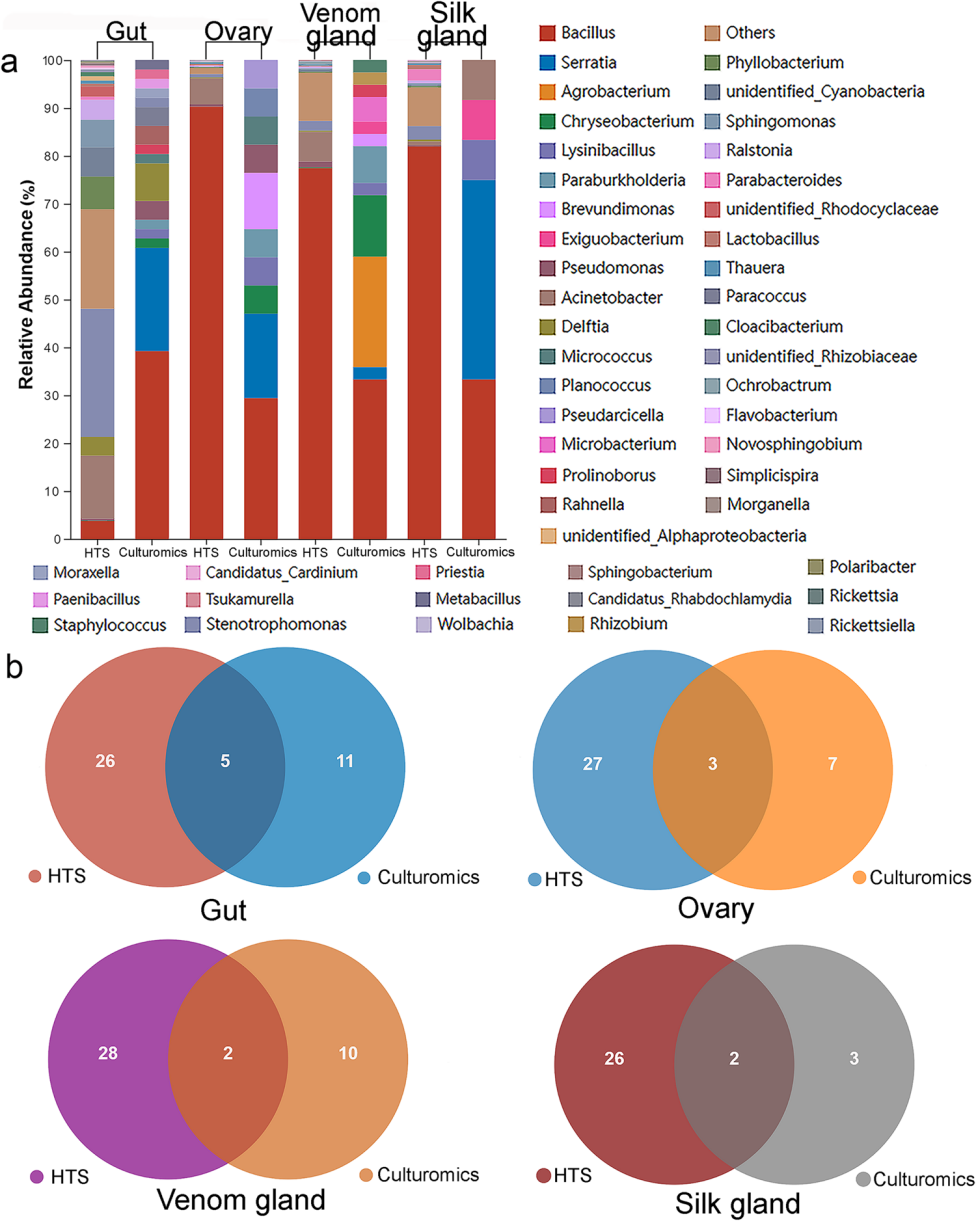
Comparison of the genera detected in culturomics and the top 30 bacterial genera of HTS in different tissues. **a** Taxonomic distribution of bacteria at the genus level. **b** Venn diagram showing the overlap of genera acquired from the different methods in each tissue

At the genus level, the similarities and differences between the top 30 genera of the HTS dataset, and isolated genera from different tissues were investigated (SI Table 6). *Bacillus* exhibited relatively higher abundance in most samples, and the proportion of *Bacillus* in the gut as determined by HTS was only 3.7%. It is noteworthy that the “others” in the gut sample accounted for the largest proportion at 20.7% (Fig. 6a), indicating that the species richness of the gut microbiota was significantly higher than that of the other samples (SI Table 6).

The bacterial genera obtained from the same organization using two different methods were compared, and the results are presented in Fig. 6b. The Venn analysis revealed that six genera, *Bacillus*, *Chryseobacterium*, *Delftia*, *Pseudomonas*, *Stenotrophomonas*, and *Acinetobacter*, were common in both the culturomics and HTS groups. The first five genera were shared by the two gut samples and accounted for 35% of the total bacterial diversity detected in the HTS dataset. Three genera, *Bacillus*, *Chryseobacterium*, and *Pseudomonas*, were found in both ovary samples and collectively represented 90.8% of the HTS dataset. Two genera were identified in the same tissue samples for the bacterial communities of the venom gland and silk gland, respectively, and accounted for approximately 80% of the corresponding HTS dataset (Fig. 6b, SI Table 6).

Ten genera, including *Agrobacterium*, *Prolinoborus*, *Moraxella*, *Rahnella*, *Serratia*, *Exiguobacterium*, *Metabacillus*, *Lysinibacillus*, *Priestis*, and *Planococcus*, were exclusively isolated using the culturomics approach and were not detected in the HTS dataset. This suggests that their relative abundance in spider tissues may be below the detection limit of the HTS method, or the lack of detection could be due to biases in DNA extraction or amplification.

### Bioactivity Screening Assays

#### Assessment of Bifenthrin and Atrazine Degradation Capability

**Fig. 7:**
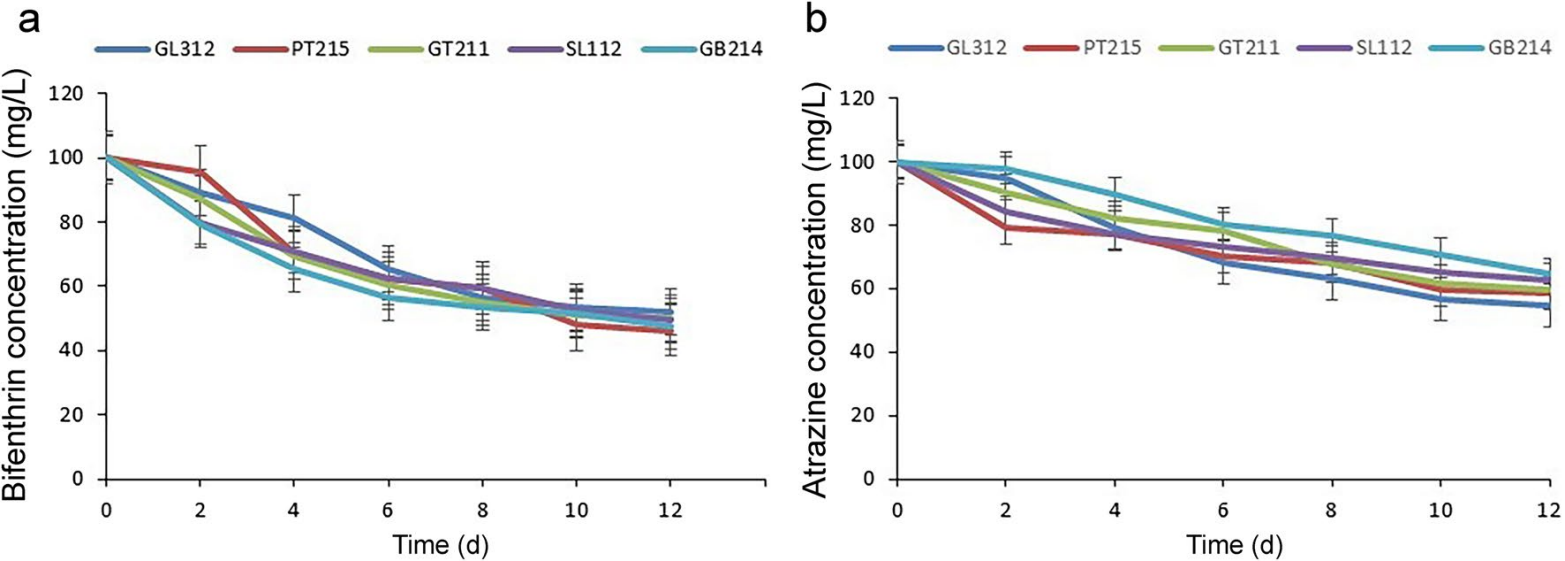
Degradation of pesticides by the five strains. **a** The degradation of bifenthrin. **b**The degradation of atrazine

The study investigated the pesticide tolerance of the isolates by adding bifenthrin and atrazine to a MSM at increasing concentrations. The results revealed that five strains, GB214 (*Plasmodiophora* *brassicae*), GL312 (*Priestia* *megaterium*), GT211 (*Bacillus* *albus*), PT215 (*Bacillus* *manliponensis*), and SL112 (*Serratia* *marcescens*), were capable of growing in a mineral salt medium containing 100 mg/L of pesticides. The degradation efficiency of these isolates was analyzed at a pesticide concentration of 100 mg/L, and it was revealed after 12 days. The degradation rates of bifenthrin were observed to be 52.4% (GB214), 47.8% (GL312), 50.1% (GT211), 53.7% (PT215), and 50.4% (SL112) after 12 days (Fig. 7a). Similarly, the degradation rates of atrazine were found to be 35.4% (GB214), 45.1% (GL312), 40.5% (GT211), 41.3% (PT215), and 37.1% (SL112) on the 12th day (Fig. 7b).

#### Antibacterial Activity

Of the 119 isolates, 28 (23.5%) demonstrated inhibitory activity against at least one indicator organism. The inhibitory activity was similar across the 11 genera tested and was highest in isolates from the venom gland. Thirteen strains were isolated from the venom gland, while eight, five, and two strains were isolated from the gut, ovary, and silk gland, respectively (Table 1). Large inhibition zones with a diameter over 15 mm were observed in seven test plates of *S. aureus*, ten test plates of *E. faecalis*, and six test plates of *A. baumanii*, respectively. Finally, strains GL103, GL312, GR215, PL211, PL111, PL213, and PL316, which showed higher antibacterial activity against at least two pathogen indicators, were chosen for cytotoxicity experiments.

**Table 1:** Antibacterial activities of the isolates

Isolates	Proposed identity	*S. aureus*	*E. faecalis*	*A. baumanii*
GL103	*Serratia marcescens*	14.1 ± 1.2	18.3 ± 1.0	16.2 ± 0.9
GL212	*Prolinoborus fasciculus*	14.8 ± 1.0	13.1 ± 1.1	14.9 ± 0.8
GL312	*Priestia megaterium*	17.0 ± 1.4	22.1 ± 1.2	19.2 ± 1.2
GL314	*Serratia marcescens*	12.0 ± 1.2	12.9 ± 1.0	13.0 ± 1.3
GT211	*B. albus*	12.8 ± 1.3	17.6 ± 0.9	14.5 ± 1.5
GT215	*Serratia marcescens*	14.3 ± 1.1	13.8 ± 1.1	12.9 ± 1.6
GT313	*Serratia marcescens*	19.1 ± 2.3	12.6 ± 1.3	14.8 ± 1.1
GR215	*Delftia tsuruhatensis*	22.2 ± 2.0	18.3 ± 1.4	13.5 ± 0.9
OL213	*Serratia marcescens*	14.2 ± 0.9	12.0 ± 1.5	12.9 ± 1.7
OL217	*Lysinibacillus fusiformis*	12.5 ± 1.0	13.6 ± 2.4	13.1 ± 1.5
OL307	*Serratia marcescens*	12.6 ± 1.9	14.1 ± 2.2	12.0 ± 1.1
OT202	*Serratia marcescens*	14.8 ± 2.1	13.2 ± 0.8	12.8 ± 1.2
OT306	*Pseudomonas oryzihabitans*	19.3 ± 1.2	13.5 ± 1.3	12.9 ± 1.4
PB221	*B. albus*	12.1 ± 1.1	16.6 ± 1.2	14.1 ± 1.4
PL116	*Microbacterium proteolyticum*	13.9 ± 1.3	13.0 ± 1.4	13.5 ± 0.9
PL118	*Agrobacterium deltaense*	13.1 ± 1.9	12.0 ± 0.9	14.8 ± 1.3
PL211	*B. zanthoxyli*	16.5 ± 2.6	25.3 ± 1.2	18.6 ± 1.8
PL111	*B. albus*	13.9 ± 1.4	17.1 ± 1.1	18.1 ± 0.3
PL213	*B. wiedmannii*	14.6 ± 1.4	16.0 ± 1.8	18.2 ± 1.0
PL214	*Brevundimonas vesicularis*	13.7 ± 0.5	14.5 ± 1.9	12.9 ± 1.5
PL217	*Chryseobacterium aquaticu*	12.0 ± 1.1	12.3 ± 2.1	12.6 ± 0.5
PL218	*Agrobacterium deltaense*	14.1 ± 0.9	14.0 ± 0.6	13.2 ± 1.6
PL219	*Lysinibacillus fusiformis*	14.3 ± 0.9	18.3 ± 1.1	13.1 ± 1.2
PL315	*Serratia marcescens*	13.5 ± 1.5	13.5 ± 1.5	13.0 ± 0.9
PL316	*B. cereus*	23.2 ± 1.6	18.5 ± 1.4	19.0 ± 1.1
PT215	*B. manliponensis*	14.5 ± 1.8	12.1 ± 1.3	12.0 ± 1.5
SL212	*B. paramycoides*	19.0 ± 1.7	13.5 ± 1.2	13.0 ± 1.0
SL213	*Serratia marcescens*	13.1 ± 1.2	12.0 ± 1.1	14.5 ± 1.2

#### Anti-gastric Cancer Cell Activity

**Fig. 8:**
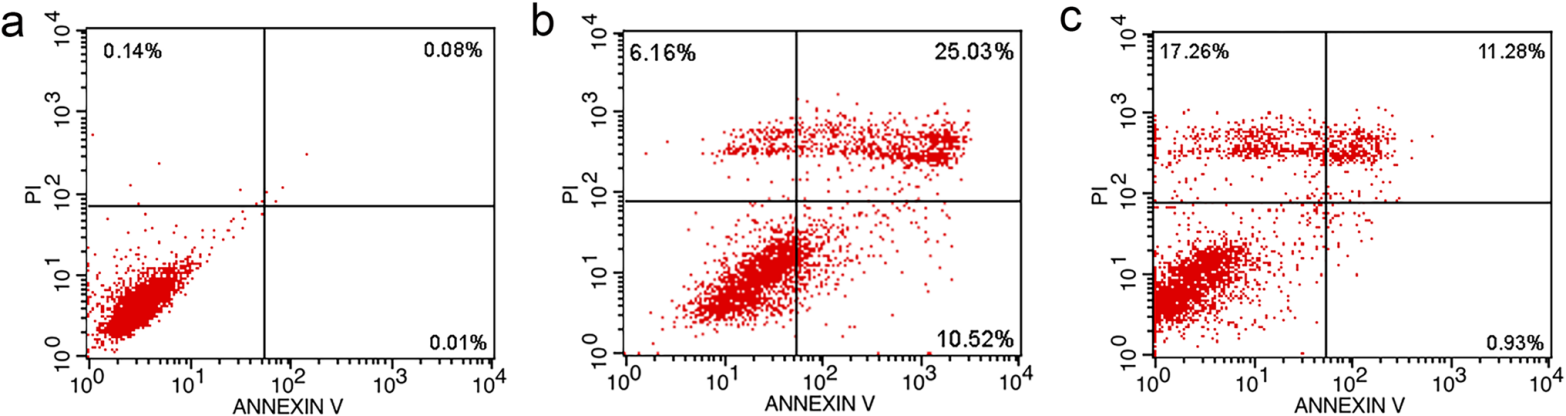
Apoptotic cells in MGC-803 cells induced by extracts of strain PL316. **a** Flow cytometry of untreated cells. **b** Apoptotic cells under the exposure of 12 h. **c** Apoptotic cells under the exposure of 36 h

The study assessed the antitumor activity of various strains against the MGC-803 human gastric cancer cell line. Strains GL312 (*Priestia megaterium*) and PL211 (*B. zanthoxyli*) exhibited strong cytotoxicity, with IC_50_ values of 5.188 µg/mL and 5.134 µg/mL for the crude extract, slightly higher than the IC_50_ value of 3.906 µg/mL for the positive control taxol. Strain PL316 demonstrated the most potent cytotoxic effect against MGC-830 cells, with an IC_50_ value of 2.331 µg/mL, and was chosen for the apoptosis experiment. The flow cytometry results indicated that the crude extract of PL316 fermentation broth induced apoptosis in MGC-803 cells, and the death rate gradually increased as the treatment time extended (Fig. 8).

## Discussion

Investigations into the bacteria associated with spiders have primarily employed next-generation sequencing, yielding a significant amount of information on community structure [4, 35], function [6], temporal and spatial dynamics [36], phylosymbiosis [2], and distribution preference [7, 8]. In this study, our aim was to comprehend the microbial community of spiders by integrating HTS and culturomics to explore the diversity of *H. venatoria* microbes and their functions.

### Diversity of Tissue-Associated Bacteria

It was reported that Proteobacteria and Firmicutes were the most dominant phyla in the spider gut [6, 11] and in other arthropods [37]. Tyagi et al. (2021) revealed *Acinetobacter* as the predominant genus of gut bacteria in certain spider species [38]. These sequencing data provide valuable insights into the diverse microbial community of spiders. Previous studies have focused on the endosymbiotic interactions between specific genera of bacteria and host reproductive mechanisms, particularly *Wolbachia*, *Rickettsia*, *Cardinium*, and *Spiroplasma* [39]. They found that *Rickettsia* and *Wolbachia* were the most prevalent endosymbionts, even in the spider’s gut [38, 40]. However, in the present study, these two genera were not found to be predominant in the bacterial community of all samples. *Wolbachia* was present in relatively low abundance, ranging from 0.01% in the venom gland to 0.1% in the ovary tissue (SI Table 6), which may be related to its well-known ability to manipulate the reproductive biology of the host [41].

Among the four tissues, *Acinetobacter* had the highest average relative abundance in the ovary. Additionally, *Acinetobacter* was the dominant microbiota in the eggs and first instar larvae of *Henosepilachna vigintioctopunctata* [42] and *Cnaphalocrocis medinalis* [43]. Moreover, the overall bacterial diversity in ovary tissue was relatively low in the present study, which is consistent with previous findings in the gonads of *Chorthippus parallelus*. This is in contrast to the much higher bacterial diversity reported in the fruit fly *Bactrocera minax* [44].

Spider venom shares a common origin with the digestive gland [39], and many components of spider venom demonstrate potential activities, including anticancer, antimicrobial, and analgesic effects [45, 46]. Despite a large number of studies on venom antimicrobial peptides in the past 5 years, few studies have focused on venom-microorganism interactions. In this study, 33.3% of the strains exhibiting high antibacterial activity were isolated from the venom gland, which had the tissue with the highest proportion of strains with high antibacterial activity (Table 1). Two out of the three strains that exhibited high cytotoxicity against gastric cancer were also obtained from the venom gland. The venom gland, which is a relatively enclosed and independent environment, may provide a unique microenvironment that facilitates microbial adaptation. Exploring the relationship between the microbial community and the function of venom may add another dimension to research in this area [47].

Spider silk is widely known for its exceptional biomechanical properties and biocompatibility [48]. In ancient times, Greeks and Romans used spider silk poultices as an antipyretic and hemostatic agent to treat bleeding battle wounds [49]. The present study represents the first attempt to investigate the microbial community inhabiting the silk gland of spiders. Our findings revealed that the bacterial species richness of the silk gland was similar to that of the ovary or venom gland, but the number of isolates was comparatively lower. The isolates were mainly composed of *Bacillus*, *Serratia*, *Acinetobacter*, *Lysinibacillus*, and *Exiguobacterium*.

The results of the alpha diversity analysis suggest that the Chao index serves as an indicator of the richness of bacterial communities associated with spiders, whereas the Shannon index reflects both the diversity and evenness of these spider-related bacterial communities. As illustrated in Fig. 2, no statistically significant differences were observed in the Chao index (*P* =  0.1441) and Shannon index (*P* =  0.0703) of bacteria associated with various spider tissues. However, a notable exception was found in the gut samples, which exhibited significant differences compared to other three samples. This suggests that the gut of the spider *H. venatoria* harbors a greater abundance and diversity of bacteria compared to other tissues. This phenomenon can be attributed to the fact that the gut typically hosts a greater number of microorganisms and species compared to other anatomical regions in most animals, including humans. Cazemier et al. (1997) [50] suggested that the proportion of gut-associated bacteria is higher than that of the overall microbial community in various species of arthropods. Conversely, the bacterial community structures among the other three tissues appear to be highly similar. Furthermore, NMDS analysis reinforces this finding, showing that the points representing the three samples are tightly clustered, indicating a high degree of similarity, while the gut sample is distinctively separated, highlighting significant differences in species composition. The findings from the bipartite network analysis were consistent with both α-diversity and β-diversity analyses. This consistency across diversity metrics suggests that the gut microbiota exhibits significant richness, whereas the bacterial communities within the other three tissues display greater uniformity. In total, our analysis identified 32 genera across all four tissues, with the gut harboring the highest number of unique genera, totaling 250 (SI Table 3). Notably, within the gut, the genus *Acinetobacter* was found to be the most abundant, followed by *Stenotrophomonas*. These genera have been previously identified as predominant microorganisms in various spider samples [51]. Moreover, a strain of *Stenotrophomonas* exhibiting robust feather degradation was isolated from the spider gut, further highlighting their ecological significance [52].

Among the three tissues of venom glands, silk glands, and ovaries, the genus *Bacillus* exhibits the highest abundance, possibly due to its remarkable environmental adaptability [53]. Notably, the gut-specific genera account for the most significant proportion among the three, totaling 25 genera. However, it is noteworthy that the proportion of norank genera with an undetermined taxonomic status (norank) is also the highest within these genera. Traditionally, scientists have regarded animal venom as a sterile environment, owing to its rich antibacterial properties and potent bactericidal effects. However, recent research challenges this notion, revealing the presence of microorganisms within venom and its secreting glands. Esmaeilishirazifar’s investigation into the venom organs and microbiota of five snakes and two spiders confirms the isolation of microorganisms from the venom of *Naja nigricollis* and *Poecillothera regalis* [54]. These findings suggest that the venom glands of spiders are likely a rich source of undiscovered bacteria.

Previously, Iwai employed a method of collecting spider webs by inducing spiders to traverse agar plates, subsequently isolating actinomycetes from these webs [55]. Other than this, there has been almost no other research on silk-associated bacteria. Our current study confirms the presence of bacteria inhabiting these glands, underscoring the research significance of silk glands as a distinctive microbial habitat. Furthermore, our study revealed that the ovary samples exhibited the lowest species richness and unique genera compared to other tissues. This finding is consistent with previous research indicating that bacterial communities in the gut display higher diversity and relative abundance compared to the reproductive glands of spiders [56].

### Comparative Analysis of HTS and Culturomics

Previous studies have reported limitations of metagenomics in identifying certain organisms and may even fail to detect certain culturable microorganisms [51]. In this study, the alpha diversity exhibited adequate sequencing depth. However, an interesting finding was that more than 38% of the isolated bacteria went undetected when using HTS. This observation emphasizes the limitations of the HTS method, which may be due to potential biases stemming from DNA extraction and amplification.

One potential explanation for these limitations is the inevitable selectivity of primers and variations in the length of the targeted amplification region [57]. Moreover, biases in HTS outcomes can also stem from other experiment-specific factors. For instance, the selectivity of DNA restriction enzymes and the method used to fragment DNA samples [58].

Even in the era of omics technologies, cultivation remains a fundamental practice in microbial ecology. Microbial cultures are necessary to obtain pure strains with bioengineering potential and for validating microbial metabolism. However, we must also acknowledge the biases associated with cultivation. In this study, the culturomics approach yielded lower taxonomic diversity compared to 16S rRNA gene amplicon sequencing. Only 25 genera were isolated, accounting for 5.5%, of the 458 genera detected by the HTS method. Culture media are typically designed to isolate the 13 most abundant aerobic bacterial genera, but only 6 of these were successfully isolated. It may be related to many microbial species in the biosphere that resist laboratory cultivation despite providing the chemical components of the natural environment [59]. During the process of isolating and purifying bacteria associated with spiders, researchers artificially select specific strains. Bacterial isolates with slow growth rates often require cultivation for 7–10 days, followed by 1–2 rounds of purification lasting at least 14 days. Additionally, many “viable but non-culturable” species pose additional challenges [60]. The study originally aimed to comprehensively depict the genetic diversity of bacteria associated with spiders and to guide further screening of strains with bioengineering significance by combining HTS and culturomics methods. Currently, additional exploration is needed to fully comprehend the findings of culturomics.

Interestingly, several isolated strains have been reported as sources of active compounds. The proportion of *Serratia* and *Bacillus* in the isolated strains is relatively high. Many bacteria from these two genera are known for producing active compounds, and their metabolic capacity is becoming increasingly important in antimicrobial research [13].

### Functional Analysis of Spider-Associated Bacteria

Pesticides are frequently utilized to manage pests, but they can also have adverse effects on human health and the environment. Among the most widely used pesticides are bifenthrin and atrazine, which can pose environmental problems and lead to human exposure when extensively used. Bacteria can break down organic pesticides and can be found in various ecosystems, including marine sponges [61], sewage, soil [62], and water [63]. In recent years, there has been growing interest in the role of spider-associated bacteria in insect drug resistance and detoxification metabolism [64]. Previous studies have shown that the gut microbiome can assist in detoxification by regulating the host’s immune system [65]. For example, the gut symbiont of the tephritid pest fruit fly *Bactrocera dorsalis* has been shown to enhance the host’s resistance to the organophosphate insecticide trichlorphon [66]. Furthermore, pesticide-degrading bacteria have been identified in insect hosts such as *Lepidoptera* [67], *Diptera* [66], and *Coleoptera* [68]. There is growing evidence suggesting that the gut microbiota of animals plays a significant role in pesticide-induced toxicity, indicating that the gut microbiota may inadvertently be affected by pesticides [69]. Bacteria capable of degrading bifenthrin and atrazine have mainly been identified as *Pseudomonas*, *Delftia*, and *Acinetobacter* [69]. In the present study, only GB214 was identified as belonging to the genus *Pseudomonas*. The bacteria isolated from spiders in this study were found not only to tolerate pesticide concentrations up to 500 mg/L but also to possess certain degradation abilities. However, the degradation rate observed in this study was not particularly high, in contrast to the 70–95% degradation of 50–100 mg/L pesticides reported in previous studies [70].

Research has demonstrated the extensive opportunities for discovering antimicrobial substances from insect symbionts [71]. The discovery of new antibacterial compounds depends on the separation and purification of components with antibacterial activity while tracking their activity. Bacteria isolated from the gut of cockroaches have been shown to produce a broad spectrum of antibacterial molecules, while bacteria isolated from honeybees have been found to produce phenazine-like compounds with antibacterial activities [72]. The majority of active strains belong to Enterobacterales, Bacillales, Lactobacillales, and Pseudomonadales [13]. Nazipi et al. reported isolating bacteria from the nest material of the spider (*Stegodyphus dumicola*) and identified bacterial strains with antimicrobial activity [16].

*S. aureus*, *E. faecalis*, and *A. baumanii* were chosen to conduct antibacterial experiments *in vitro*. Among the 28 active bacteria identified, there are ten strains belonging to the top 10 genera with high abundance in HTS data, including *Bacillus*, *Chryseobacterium*, and *Delftia* (only culturable strains included). There are 19 strains from the top 5 genera with high abundance in isolated strains. These five genera include *Bacillus*, *Serratia*, *Agrobacterium*, *Chryseobacterium*, and *Paraburkholderia*. Some of these genera, such as *Bacillus*, *Chryseobacterium*, and *Delftia*, have all been confirmed to have antibacterial activity [73]. Additionally, many components of spider venom have shown potential therapeutic activity against a wide range of human diseases, including cancer, microbial infections, malaria, arrhythmia, and erectile dysfunction [46]. Moreover, several components of spider venom exhibit potential bioinsecticidal activity. Even isolated microorganisms have been found in spider venom [70]. Among the active bacteria identified, the highest proportion originates from the venom glands. Further research is necessary to determine the potential relationship between these venom functions and bacteria associated with venom. In recent years, there has been a growing interest in identifying and characterizing symbiotic bacteria from various environments, such as marine environments and invertebrates, because of their potential to produce compounds with significant anticancer activity [18]. Previous studies have shown that *Actinobacteria* isolated from volcanic sea islands exhibited cytotoxic activity against Hela and A549 cell lines [33]. Additionally, an endophytic bacterium isolated from the medicinal plant *Ophiopogon japonicas*, identified as *Bacillus amyloliquefaciens*, also demonstrated antitumoral activity [74]. In this study, three bacteria strains, namely GL312, PL211, and PL316 isolated from the gut and venom gland. They were identified as belonging to the genus *Bacillus* (PL211 and PL316) and *Priestia* (GL312). These strains were found to exhibit cytotoxic activity against cancer cells.

Interestingly, *Bacillus* accounts for the highest abundance in both HTS and culturomics solutions, suggesting that* Bacillus* has greater environmental adaptability and can grow in a wide range of environments [53]. The study also exhibited significant antibacterial and anti-tumor activity. Previous research has demonstrated that specific *Bacillus* strains, including *Bacillus amyloliquefaciens*, *Bacillus cereus*, and *Bacillus subtilis*, exhibit anticancer activity [75–77]. *Bacillus thuringiensis* (Bt), a widely recognized biopesticide in the market, has also been found to exhibit specific activity against various human cancer cell lines [78]. The parasporin produced by *B. thuringiensis* has been found to exhibit cytotoxic effects on cancer cells while remaining non-toxic to peripheral blood mononuclear cells [79]. These findings suggest that *Bacillus* may be a promising source of bioactive compounds for anticancer drug development and require further investigation.

## Conclusion and Perspectives

This study focuses on the bacteria associated with *H. venatoria* and conducts in-depth research on bacterial communities associated with different tissues with function verification of some isolates. HTS was performed to clarify the bacterial community structure and acquired 119 isolates using culturomics. Functional tests demonstrated that these spider-associated bacteria were capable of producing a variety of secondary metabolites with potential biological activity. Among them, 5 strains exhibited degradation efficiency of bifenthrin and atrazine, 28 strains displayed antibacterial activity, and 3 strains showed significant cytotoxicity against the human gastric cancer cell line MGC-803.

This research holds significant importance for the development of biotechnological resources aimed at cultivating pure cultures of bacteria associated with arthropods. By studying the functions of these bacteria, we can explore their potential applications in the biomedical and agricultural fields.

### Supplementary Information

Below is the link to the electronic supplementary material.

Supplementary file1 (DOCX 106 KB)

## Data Availability

The raw data of all samples generated in present work is available in the Sequence Read Archive (SRA, National Center for Biotechnology Information) under the accession number SAMN27968563 to SAMN27968574.

## References

[1] World Spider Catalog (2023) World Spider Catalog Version 23.5.,. http://wsc.nmbe.ch, accessed on (Jan 18, 2023)

[2] Dunaj SJ, Bettencourt BR, Garb JE, Brucker RM (2020). Spider phylosymbiosis: divergence of widow spider species and their tissues’ microbiomes. BMC Evol Biol.

[3] Tyagi K, Tyagi I, Kumar V (2021). Insights into the gut bacterial communities of spider from wild with no evidence of phylosymbiosis. Saudi J Biol Sci.

[4] Hu G, Zhang L, Yun Y, Peng Y (2019). Taking insight into the gut microbiota of three spider species: no characteristic symbiont was found corresponding to the special feeding style of spiders. Ecol Evol.

[5] Kumar V, Tyagi I, Tyagi K, Chandra K (2020). Diversity and structure of bacterial communities in the gut of spider: Thomisidae and Oxyopidae. Front Ecol Evol.

[6] Wu R, Wang L, Xie J, Zhang Z (2021). Diversity and function of wolf spider gut microbiota revealed by shotgun metagenomics. Front Microbiol.

[7] Zhang W, Liu F, Zhu Y (2021). Differing dietary nutrients and diet-associated bacteria has limited impact on spider gut microbiota composition. Microorganisms.

[8] Gao Y, Wu P, Cui S, et al (2022) Divergence in gut bacterial community between females and males in the wolf spider *Pardosa astrigera*. Ecol Evol 12:. 10.1002/ece3.8823 10.1002/ece3.8823 PMC9005928 35432934

[9] Lee SA, Park J, Chu B (2016). Comparative analysis of bacterial diversity in the rhizosphere of tomato by culture-dependent and -independent approaches. J Microbiol.

[10] Pereira AC, Bandeira V, Fonseca C, Cunha MV (2020). Crosstalk between culturomics and microbial profiling of Egyptian mongoose (Herpestes ichneumon) gut microbiome. Microorganisms.

[11] Vartoukian SR (2016). Cultivation strategies for growth of uncultivated bacteria. J Oral Biosci.

[12] Durán-Viseras A, Andrei A, Vera-Gargallo B (2021). Culturomics-based genomics sheds light on the ecology of the new haloarchaeal genus *Halosegnis*. Environ Microbiol.

[13] Van Moll L, De Smet J, Cos P, Van Campenhout L (2021). Microbial symbionts of insects as a source of new antimicrobials: a review. Crit Rev Microbiol.

[14] Shao M-W, Lu Y-H, Miao S (2015). Diversity, bacterial symbionts and antibacterial potential of gut-associated fungi isolated from the Pantala flavescens Larvae in China. PLoS ONE.

[15] Viju N, Punitha SMJ, Satheesh S (2021). An analysis of biosynthesis gene clusters and bioactivity of marine bacterial symbionts. Curr Microbiol.

[16] Nazipi S, Elberg CL, Busck MM (2021). The bacterial and fungal nest microbiomes in populations of the social spider Stegodyphus dumicola. Syst Appl Microbiol.

[17] Liu J, Zhang M, Zhang R (2016). Comparative studies of the composition of bacterial microbiota associated with the ruminal content, ruminal epithelium and in the faeces of lactating dairy cows. Microb Biotechnol.

[18] Yao H, Sun X, He C (2019). Phyllosphere epiphytic and endophytic fungal community and network structures differ in a tropical mangrove ecosystem. Microbiome.

[19] Ren Y, Xun W, Yan H (2020). Functional compensation dominates the assembly of plant rhizospheric bacterial community. Soil Biol Biochem.

[20] Oberhardt MA, Zarecki R, Gronow S (2015). Harnessing the landscape of microbial culture media to predict new organism–media pairings. Nat Commun.

[21] Xu J, Sun L, Xing X (2020). Culturing bacteria from fermentation pit muds of Baijiu with culturomics and amplicon-based metagenomic approaches. Front Microbiol.

[22] Button DK, Schut F, Quang P (1993). Viability and isolation of marine bacteria by dilution culture: theory, procedures, and initial results. Appl Environ Microbiol.

[23] Sarhan MS, Hamza MA, Youssef HH (2019). Culturomics of the plant prokaryotic microbiome and the dawn of plant-based culture media – a review. J Adv Res.

[24] Thompson J (1997). The CLUSTAL_X windows interface: flexible strategies for multiple sequence alignment aided by quality analysis tools. Nucleic Acids Res.

[25] Kumar S, Stecher G, Li M (2018). MEGA X: molecular evolutionary genetics analysis across computing platforms. Mol Biol Evol.

[26] Sharma A, Kalyani P, Trivedi VD, et al (2019) Nitrogen-dependent induction of atrazine degradation pathway in *Pseudomonas* sp. strain AKN5. FEMS Microbiol Lett 366:. 10.1093/femsle/fny277 10.1093/femsle/fny277 30500940

[27] Chen S, Luo J, Hu M (2012). Microbial detoxification of bifenthrin by a novel yeast and its potential for contaminated soils treatment. PLoS ONE.

[28] Enayati EA, Vontas JG, Small G (2001). Hemingway J. Quantification of pyrethroid insecticides from treated bednets using a mosquito recombinant glutathione S-transferase. Med Vet Entomol.

[29] Moreira AJ, Lemos SG, Coelho D (2022). UV–Vis spectrophotometry coupled to chemometric analysis for the performance evaluation of atrazine photolysis and photocatalysis. Environ Sci Pollut Res.

[30] Husain DR, Wardhani R (2021) Antibacterial activity of endosymbiotic bacterial compound from Pheretima sp. earthworms inhibit the growth of Salmonella Typhi and Staphylococcus aureus: in vitro and in silico approach. Iran J Microbiol 10.18502/ijm.v13i4.6981 10.18502/ijm.v13i4.6981 PMC8421580 34557283

[31] Marcolefas E, Leung T, Okshevsky M (2019). Culture-dependent bioprospecting of bacterial isolates from the Canadian high arctic displaying antibacterial activity. Front Microbiol.

[32] Cui P, Kong K, Yao Y (2022). Community composition, bacterial symbionts, antibacterial and antioxidant activities of honeybee-associated fungi. BMC Microbiol.

[33] Wang L, Peng C, Gong B (2022). Actinobacteria community and their antibacterial and cytotoxic activity on the Weizhou and Xieyang Volcanic Islands in the Beibu Gulf of China. Front Microbiol.

[34] Ran X, Zhang G, Li S, Wang J (2017). Characterization and antitumor activity of camptothecin from endophytic fungus *Fusarium solani* isolated from *Camptotheca acuminate*. Afr Health Sci.

[35] Busck MM, Settepani V, Bechsgaard J (2020). Microbiomes and specific symbionts of social spiders: compositional patterns in host species, populations, and nests. Front Microbiol.

[36] Busck MM, Lund MB, Bird TL (2022). Temporal and spatial microbiome dynamics across natural populations of the social spider *Stegodyphus dumicola*. FEMS Microbiol Ecol.

[37] Chen B, Teh B-S, Sun C (2016). Biodiversity and activity of the gut microbiota across the life history of the insect herbivore Spodoptera littoralis. Sci Rep.

[38] Tyagi K, Tyagi I, Kumar V (2021). Interspecific variation and functional traits of the gut microbiome in spiders from the wild: the largest effort so far. PLoS ONE.

[39] Valladão R, Neto OBS, de Oliveira GM (2023). Digestive enzymes and sphingomyelinase D in spiders without venom (Uloboridae). Sci Rep.

[40] Nasir M, Zhao C, Luo J (2021). Population dynamics, hunting nature on insect pests and existence of symbiotic bacterial microbes among leading transgenic cotton spiders. J Asia-Pac Entomol.

[41] Rowley SM, Raven RJ, McGraw EA (2004) Wolbachia pipientis in Australian Spiders. Curr Microbiol 49:. 10.1007/s00284-004-4346-z 10.1007/s00284-004-4346-z 15386106

[42] Li H, Zhao C, Yang Y (2021). The influence of gut microbiota on the fecundity of *Henosepilachna vigintioctopunctata* (Coleoptera: Coccinellidae). J Insect Sci.

[43] Yajun Yang, Xiaogai Liu, Hongxing Xu, Yinghong Liu (2020) The abundance and diversity of gut bacteria of rice leaffolder Cnaphalocrocis medinalis (Guenée) across life stages. 23:430–438 10.1016/j.aspen.2020.03.006

[44] Wang A, Yao Z, Zheng W, Zhang H (2014). Bacterial communities in the gut and reproductive organs of Bactrocera minax (Diptera: Tephritidae) Based on 454 Pyrosequencing. PLoS ONE.

[45] Santos DM, Verly RM, Piló-Veloso D (2010). LyeTx I, a potent antimicrobial peptide from the venom of the spider Lycosa erythrognatha. Amino Acids.

[46] Akef HM (2018). Anticancer, antimicrobial, and analgesic activities of spider venoms. Toxicol Res.

[47] Ul-Hasan S, Rodríguez-Román E, Reitzel AM (2019). The emerging field of venom-microbiomics for exploring venom as a microenvironment, and the corresponding initiative for venom associated microbes and parasites (iVAMP). Toxicon X.

[48] Franco AR, Fernandes EM, Rodrigues MT (2019). Antimicrobial coating of spider silk to prevent bacterial attachment on silk surgical sutures. Acta Biomater.

[49] Salehi S, Koeck K, Scheibel T (2020). Spider silk for tissue engineering applications. Molecules.

[50] Cazemier AE, Hackstein JHP, den Camp HJMO (1997). Bacteria in the intestinal tract of different species of arthropods. Microb Ecol.

[51] Yashiro E, Spear RN, McManus PS (2011). Culture-dependent and culture-independent assessment of bacteria in the apple phyllosphere: apple phyllosphere bacteria. J Appl Microbiol.

[52] Qu F, Chen Q, Ding Y (2018). Isolation of a feather-degrading strain of bacterium from spider gut and the purification and identification of its three key enzymes. Mol Biol Rep.

[53] Earl AM, Losick R, Kolter R (2008). Ecology and genomics of Bacillus subtilis. Trends Microbiol.

[54] Esmaeilishirazifard E, Usher L, Trim C (2022). Bacterial adaptation to venom in snakes and Arachnida. Microbiol Spectr.

[55] Iwai K, Iwamoto S, Aisaka K, Suzuki M (2009). Isolation of novel actinomycetes from spider materials. Actinomycetologica.

[56] Liu Y, Liu J, Zhang X, Yun Y (2023). Diversity of bacteria associated with guts and gonads in three spider species and potential transmission pathways of microbes within the same spider host. Insects.

[57] Wang JR, Quach B, Furey TS (2017). Correcting nucleotide-specific biases in high-throughput sequencing data. BMC Bioinformatics.

[58] Geisen S, Laros I, Vizcaíno A (2015). Not all are free-living: high-throughput DNA metabarcoding reveals a diverse community of protists parasitizing soil metazoa. Mol Ecol.

[59] Prakash O, Parmar M, Vaijanapurkar M (2021). Recent trend, biases and limitations of cultivation-based diversity studies of microbes. FEMS Microbiol Lett.

[60] Wideman NE, Oliver JD, Crandall PG, Jarvis NA (2021). Detection and potential virulence of viable but non-culturable (VBNC) Listeria monocytogenes: a review. Microorganisms.

[61] Ortega SN, Nitschke M, Mouad AM (2011). Isolation of Brazilian marine fungi capable of growing on DDD pesticide. Biodegradation.

[62] Zhang J, Liang S, Wang X (2019). Biodegradation of Atrazine by the Novel *Klebsiella variicola* Strain FH-1. BioMed Res Int.

[63] Cáceres TP, Megharaj M, Naidu R (2008). Biodegradation of the pesticide fenamiphos by ten different species of green algae and cyanobacteria. Curr Microbiol.

[64] Siddiqui JA, Khan MM, Bamisile BS (2022). Role of insect gut microbiota in pesticide degradation: a review. Front Microbiol.

[65] Xia X, Sun B, Gurr GM (2018). Gut microbiota mediate insecticide resistance in the diamondback moth, Plutella xylostella (L.). Front Microbiol.

[66] Cheng D, Guo Z, Riegler M (2017). Gut symbiont enhances insecticide resistance in a significant pest, the oriental fruit fly Bactrocera dorsalis (Hendel). Microbiome.

[67] Ramya SL, Venkatesan T, Srinivasa Murthy K (2016). Detection of carboxylesterase and esterase activity in culturable gut bacterial flora isolated from diamondback moth, Plutella xylostella (Linnaeus), from India and its possible role in indoxacarb degradation. Braz J Microbiol.

[68] Akami M, Njintang NY, Gbaye OA (2019). Gut bacteria of the cowpea beetle mediate its resistance to dichlorvos and susceptibility to Lippia adoensis essential oil. Sci Rep.

[69] Singh B, Singh K, Singh G (2016). Microbial degradation of herbicides. Crit Rev Microbiol.

[70] Chen S, Hu W, Xiao Y (2012). Degradation of 3-phenoxybenzoic acid by a Bacillus sp. PLoS ONE.

[71] Chevrette MG, Carlson CM, Ortega HE (2019). The antimicrobial potential of Streptomyces from insect microbiomes. Nat Commun.

[72] Akbar N, Siddiqui R, Iqbal M (2018). Gut bacteria of cockroaches are a potential source of antibacterial compound(s). Lett Appl Microbiol.

[73] Tavarideh F, Pourahmad F, Nemati M (2022) Diversity and antibacterial activity of endophytic bacteria associated with medicinal plant Scrophularia striata. Vet Res Forum 13:. 10.30466/vrf.2021.529714.3174 10.30466/vrf.2021.529714.3174 PMC9548236 36320307

[74] Chen Y-T, Yuan Q, Shan L-T (2013). Antitumor activity of bacterial exopolysaccharides from the endophyte Bacillus amyloliquefaciens sp. isolated from Ophiopogon japonicus. Oncol Lett.

[75] Seo H-R, Kim J-Y, Kim J-H, Park K-Y (2009). Identification of *Bacillus cereus* in a Chungkukjang that showed high anticancer effects against AGS human gastric adenocarcinoma cells. J Med Food.

[76] Zhao M-F, Liang G-D, Zhou Y-J (2020). Novel Bacillus strains from the human gut exert anticancer effects on a broad range of malignancy types. Invest New Drugs.

[77] Singh N, Tapader R, Chatterjee S (2022). Subtilisin from Bacillus amyloliquefaciens induces apoptosis in breast cancer cells through ubiquitin-proteasome-mediated tubulin degradation. Int J Biol Macromol.

[78] Santos EN, Menezes LP, Dolabella SS (2022). Bacillus thuringiensis: From biopesticides to anticancer agents. Biochimie.

[79] Borin DB, Castrejón-Arroyo K, Cruz-Nolasco A (2021). Parasporin A13–2 of Bacillus thuringiensis Isolates from the Papaloapan Region (Mexico) Induce a cytotoxic effect by late apoptosis against breast cancer cells. Toxins.

