# Supplementary material for: Diversity and Functionality of Bacteria Associated with Different Tissues of Spider Heteropoda venatoria Revealed through Integration of High-Throughput Sequencing and Culturomics Approaches
Source: Microb Ecol. 2024 May 4;87:67. doi: 10.1007/s00248-024-02383-2 (PMC11069485; doi:10.1007/s00248-024-02383-2)
Supplement: Supplementary file 1 [file 248_2024_2383_MOESM1_ESM.docx]

**SI Tab.1 List of relative abundance of top 30 generain the tissues of HTS of *H. venatoria***

| Taxonomy | S1 | S2 | S3 | V1 | V2 | V3 | O1 | O2 | O3 | G1 | G2 | G3 |
| --- | --- | --- | --- | --- | --- | --- | --- | --- | --- | --- | --- | --- |
| *Acinetobacter** | 0.010402844 | 0.00542654 | 0.008672986 | 0.057227488 | 0.061232227 | 0.066255924 | 0.032393365 | 0.109241706 | 0.021492891 | 0.27464455 | 0.107606635 | 0.012440758 |
| *Bacillus** | 0.945450237 | 0.939170616 | 0.57443128 | 0.805189573 | 0.829052133 | 0.689075829 | 0.93464455 | 0.826042654 | 0.947962085 | 0.08992891 | 0.017037915 | 0.006611374 |
| *Candidatus_Cardinium* | 7.11E-05 | 7.11E-05 | 0.003412322 | 4.74E-05 | 4.74E-05 | 9.48E-05 | 0 | 0 | 4.74E-05 | 2.37E-05 | 2.37E-05 | 0 |
| *Candidatus_Rhabdochlamydia* | 0.00014218 | 2.37E-05 | 0.00014218 | 2.37E-05 | 2.37E-05 | 4.74E-05 | 4.74E-05 | 7.11E-05 | 9.48E-05 | 2.37E-05 | 4.74E-05 | 0 |
| *Chryseobacterium** | 0.000165877 | 0.000118483 | 0.000165877 | 0.001635071 | 0.001184834 | 0.001848341 | 7.11E-05 | 7.11E-05 | 4.74E-05 | 0.00021327 | 0.00014218 | 0.000402844 |
| *Cloacibacterium* | 2.37E-05 | 4.74E-05 | 0.00035545 | 0 | 0 | 0.00056872 | 7.11E-05 | 0.00042654 | 0 | 0.00056872 | 0.024905213 | 9.48E-05 |
| *Delftia** | 0.0007109 | 0.001398104 | 0.00943128 | 0.000758294 | 0.00063981 | 0.007274882 | 0.001990521 | 0.002748815 | 0.000876777 | 0.044976303 | 0.023009479 | 0.049454976 |
| *Flavobacterium** | 0.000971564 | 0.001018957 | 0.001540284 | 0.000758294 | 0.000260664 | 0.001540284 | 0.001042654 | 0.000924171 | 0.001421801 | 0.001421801 | 0.002440758 | 0.006777251 |
| *Lactobacillus* | 0.001848341 | 0.002772512 | 0.022322275 | 0.000663507 | 0.00035545 | 0.002464455 | 0.001895735 | 0.003127962 | 0.002914692 | 0.001303318 | 0.005663507 | 0.011161137 |
| *Morganella* | 0 | 2.37E-05 | 0.004004739 | 0.00014218 | 0 | 0.000260664 | 4.74E-05 | 0.000379147 | 2.37E-05 | 9.48E-05 | 0.000545024 | 0.006895735 |
| *Novosphingobium** | 0 | 2.37E-05 | 4.74E-05 | 0.000260664 | 7.11E-05 | 0.00021327 | 2.37E-05 | 0.000165877 | 0 | 0.001682464 | 0.009075829 | 0.002914692 |
| *Ochrobactrum** | 0.00035545 | 0.000189573 | 0.000308057 | 0.006540284 | 0.003436019 | 0.006492891 | 0.000592417 | 0.002535545 | 0.000260664 | 0.000331754 | 2.37E-05 | 4.74E-05 |
| *Others* | 0.028009479 | 0.029028436 | 0.185308057 | 0.101563981 | 0.076729858 | 0.123459716 | 0.007274882 | 0.020189573 | 0.00985782 | 0.057677725 | 0.282677725 | 0.283270142 |
| *Parabacteroides** | 0.000189573 | 0.001374408 | 0.069028436 | 4.74E-05 | 0 | 0.001327014 | 0.000331754 | 0.001848341 | 0.000876777 | 0.0007109 | 0.009881517 | 0.009336493 |

**SI Tab.1 Continued**

| Taxonomy | S1 | S2 | S3 | V1 | V2 | V3 | O1 | O2 | O3 | G1 | G2 | G3 |
| --- | --- | --- | --- | --- | --- | --- | --- | --- | --- | --- | --- | --- |
| *Phyllobacterium** | 7.11E-05 | 0.001208531 | 0.007037915 | 0.001563981 | 0.000687204 | 0.004478673 | 0.002606635 | 0.001279621 | 0.000971564 | 0.082369668 | 0.037914692 | 0.084336493 |
| *Polaribacter* | 0 | 0 | 0 | 0 | 0 | 2.37E-05 | 0 | 2.37E-05 | 0 | 2.37E-05 | 9.48E-05 | 0.0063507 |
| *Pseudomonas** | 0.002654028 | 0.001303318 | 0.002725118 | 0.000853081 | 0.017061611 | 0.019526066 | 0.002772512 | 0.00936019 | 0.002085308 | 0.01049763 | 0.002417062 | 7.11E-05 |
| *Ralstonia** | 0.001303318 | 0.002251185 | 0.013459716 | 0.001990521 | 0.001587678 | 0.007962085 | 0.001943128 | 0.002037915 | 0.001563981 | 0.056374408 | 0.03014218 | 0.038104265 |
| *Rickettsia* | 0.00014218 | 9.48E-05 | 0.000189573 | 4.74E-05 | 7.11E-05 | 0.000236967 | 7.11E-05 | 0.000236967 | 0.00021327 | 0.004075829 | 0.00028436 | 0.00035545 |
| *Rickettsiella* | 0.00021327 | 0.00042654 | 0.000781991 | 0.000260664 | 0.000308057 | 0.000450237 | 0.00028436 | 0.00042654 | 0.000473934 | 0.000616114 | 0.000995261 | 0.00056872 |
| *Simplicispira* | 7.11E-05 | 0.00014218 | 0.000260664 | 7.11E-05 | 2.37E-05 | 0.000189573 | 0.000189573 | 0.000260664 | 0.000118483 | 0.000118483 | 0.008317536 | 0.002962085 |
| *Sphingobacterium* | 0 | 0 | 0 | 0.000402844 | 0.000236967 | 0.000118483 | 7.11E-05 | 0.00028436 | 2.37E-05 | 0 | 2.37E-05 | 2.37E-05 |
| *Sphingomonas** | 0.000402844 | 0.001255924 | 0.009454976 | 0.001018957 | 0.000402844 | 0.005663507 | 0.000947867 | 0.000900474 | 0.00049763 | 0.05964455 | 0.051943128 | 0.060995261 |
| *Stenotrophomonas** | 0.003601896 | 0.008388626 | 0.072914692 | 0.015450237 | 0.005094787 | 0.040094787 | 0.006966825 | 0.01063981 | 0.003696682 | 0.247369668 | 0.268412322 | 0.287322275 |
| *Thauera** | 0.001895735 | 0.002156398 | 0.003696682 | 0.002630332 | 0.000900474 | 0.003791469 | 0.002630332 | 0.002677725 | 0.003672986 | 0.003957346 | 0.006255924 | 0.009194313 |

**SI Tab.1 Continued**

| Taxonomy | S1 | S2 | S3 | V1 | V2 | V3 | O1 | O2 | O3 | G1 | G2 | G3 |
| --- | --- | --- | --- | --- | --- | --- | --- | --- | --- | --- | --- | --- |
| *Tsukamurella* | 0.000118483 | 0.00014218 | 0.00021327 | 0.00056872 | 0.000308057 | 0.000308057 | 0.000189573 | 0.000165877 | 0.000165877 | 0.000165877 | 0.000260664 | 0.00028436 |
| *unidentified_Alphaproteobacteria* | 0 | 0 | 0.000165877 | 0 | 0 | 7.11E-05 | 0 | 4.74E-05 | 0 | 0.000118483 | 0.00056872 | 0.026137441 |
| *unidentified_Cyanobacteria* | 0.001066351 | 0.001090047 | 0.004146919 | 0.000118483 | 4.74E-05 | 0.013744076 | 0.000545024 | 0.000829384 | 0.00042654 | 0.047085308 | 0.056800948 | 0.07936019 |
| *unidentified_Rhizobiaceae* | 2.37E-05 | 0.00049763 | 0.00542654 | 0 | 0 | 0.002037915 | 0 | 9.48E-05 | 0 | 0.012938389 | 0.002298578 | 0.001824645 |
| *unidentified_Rhodocyclaceae* | 2.37E-05 | 7.11E-05 | 4.74E-05 | 4.74E-05 | 0.000118483 | 0.000189573 | 0.000118483 | 0.00028436 | 0.000118483 | 0.000805687 | 0.050047393 | 0.012511848 |
| *Wolbachia* | 7.11E-05 | 0.00028436 | 0.000308057 | 0.000118483 | 0.000118483 | 0.000189573 | 0.000236967 | 0.002677725 | 9.48E-05 | 0.000236967 | 0.00014218 | 0.000189573 |

*: 14 aerobic or aerotolerant bacterial genera over 0.05% in average abundance of all samples based on the HTS results of the 16S rRNA gene.

**SI Tab. 2 Number of reads for individual samples**

| #Sample_name | AW1 | AW2 | AW3 | SG1 | SG2 | SG3 | PG1 | PG2 | PG3 | OC1 | OC2 | OC3 | G1 | G2 | G3 |
| --- | --- | --- | --- | --- | --- | --- | --- | --- | --- | --- | --- | --- | --- | --- | --- |
| Raw_reads(#) | 82566 | 80192 | 80030 | 80090 | 80153 | 80120 | 83016 | 85294 | 80117 | 80172 | 80165 | 84652 | 80103 | 80090 | 80057 |
| Clean_Reads(#) | 80144 | 80192 | 80030 | 80090 | 80153 | 80120 | 80068 | 80133 | 80117 | 80172 | 80165 | 84652 | 80103 | 80090 | 80057 |

SI Table. 3 Listing of venom gland (V) specific microbiota

| Taxa | Tissues specific | Mean |
| --- | --- | --- |
| *Bacillus* | G:O:V:S (common) | 12336 |
| *Stenotrophomonas* | G:O:V:S (common) | 1693.75 |
| *Acinetobacter* | G:O:V:S (common) | 1432.75 |
| *Comamonadaceae* | G:O:V:S (common) | 445.8333 |
| *Phyllobacterium* | G:O:V:S (common) | 406 |
| *Enterobacteriaceae* | G:O:V:S (common) | 237.8333 |
| *Ralstonia* | G:O:V:S (common) | 229.0833 |
| *Delftia* | G:O:V:S (common) | 215.4167 |
| *Sphingomonas* | G:O:V:S (common) | 192.75 |
| *Parabacteroides* | G:O:V:S (common) | 173.25 |
| *Pseudomonas* | G:O:V:S (common) | 153.3333 |
| *Lactobacillus* | G:O:V:S (common) | 111.4167 |
| *Burkholderiales* | G:O:V:S (common) | 101.4167 |
| *Escherichia-Shigella* | G:O:V:S (common) | 99.58333 |
| *Thauera* | G:O:V:S (common) | 73.91667 |
| *Allorhizobium-Neorhizobium-Pararhizobium-Rhizobium* | G:O:V:S (common) | 68.33333 |
| *Cloacibacterium* | G:O:V:S (common) | 55.5 |
| *Ochrobactrum* | G:O:V:S (common) | 47.41667 |
| unclassified_k__norank_d__Bacteria | G:O:V:S (common) | 43.16667 |
| norank_f__Muribaculaceae | G:O:V:S (common) | 41.66667 |
| *Flavobacterium* | G:O:V:S (common) | 36.75 |
| *Brevundimonas* | G:O:V:S (common) | 31.25 |
| unclassified_f__Rhodobacteraceae | G:O:V:S (common) | 26.58333 |
| *Parasutterella* | G:O:V:S (common) | 17.75 |
| *Bacteroides* | G:O:V:S (common) | 17.25 |
| *Paracoccus* | G:O:V:S (common) | 14.25 |
| *Rickettsia* | G:O:V:S (common) | 12.33333 |
| *Wolbachia* | G:O:V:S (common) | 8.416667 |
| *Rickettsiella* | G:O:V:S (common) | 8.083333 |
| *Rhodococcus* | G:O:V:S (common) | 5.083333 |
| *Tsukamurella* | G:O:V:S (common) | 3.166667 |
| unclassified_o__Bacteroidales | G:O:V:S (common) | 2.916667 |
| AAP99 | V | 0.666667 |
| *Microbacterium* | V | 0.5 |
| *Paenarthrobacter* | V | 0.416667 |

SI Table.3 Continued

| Taxa | Tissues specific | Mean |
| --- | --- | --- |
| *Leucobacter* | V | 0.416667 |
| *Leuconostoc* | V | 0.416667 |
| norank_f__A4b | V | 0.416667 |
| norank_f__norank_o__IMCC26256 | V | 0.333333 |
| norank_f__norank_o__norank_c__norank_p__Zixibacteria | V | 0.333333 |
| unclassified_o__Peptostreptococcales-*Tissierellales* | V | 0.333333 |
| *Elizabethkingia* | V | 0.25 |
| norank_f__norank_o__*Ardenticatenales* | V | 0.25 |
| norank_f__norank_o__DG-20 | V | 0.25 |
| norank_f__norank_o__MSBL2 | V | 0.25 |
| norank_f__norank_o__norank_c__WCHB1-81 | V | 0.25 |
| norank_f__norank_o__norank_c__WWE3 | V | 0.25 |
| norank_f__norank_o__*Proteinivoracales* | V | 0.25 |
| *Pantoea* | V | 0.25 |
| *Roseomonas* | V | 0.25 |
| g__SEEP-SRB1 | V | 0.25 |
| *Cellulosimicrobium* | V | 0.166667 |
| norank_f__norank_o__norank_c__*Acidimicrobiia* | V | 0.166667 |
| norank_f__norank_o__S-70 | V | 0.166667 |
| *Porphyromonas* | V | 0.166667 |
| *Sediminispirochaeta* | V | 0.166667 |
| *unclassified_f__Devosiaceae* | V | 0.166667 |
| *Candidatus_Cardinium* | S | 6.333333 |
| *Bordetella* | S | 1.5 |
| Tychonema_CCAP_1459-11B | S | 1.25 |
| *Adhaeribacter* | S | 0.75 |
| norank_f__Cyclobacteriaceae | S | 0.583333 |
| Geobacillus | S | 0.416667 |
| Lachnospiraceae_NK4A136_group | S | 0.416667 |
| unclassified_f__*Alcaligenaceae* | S | 0.416667 |
| *Anaeroplasma* | S | 0.333333 |
| *Magnetospirillum* | S | 0.333333 |
| *Alloprevotella* | S | 0.25 |
| *Azoarcus* | S | 0.25 |
| *Bdellovibrio* | S | 0.25 |
| *Arenibacter* | S | 0.166667 |

SI Table.3 Continued

| Taxa | Tissues specific | Mean |
| --- | --- | --- |
| norank_f__Micavibrionaceae | S | 0.166667 |
| *Pseudahrensia* | S | 0.166667 |
| *Pseudorhodobacter* | S | 0.166667 |
| unclassified_f__Bacillaceae | S | 0.166667 |
| *Wandonia* | S | 0.166667 |
| *Caulobacter* | S | 0.083333 |
| C39 | G | 108.25 |
| *Comamonas* | G | 34.08333 |
| norank_f__NS9_marine_group | G | 24.91667 |
| norank_f__Methylococcaceae | G | 20.16667 |
| *Polaribacter* | G | 18.16667 |
| *Polynucleobacter* | G | 16.5 |
| NS5_marine_group | G | 13.5 |
| *Rhodobacter* | G | 13 |
| *Aurantisolimonas* | G | 10 |
| *Alcanivorax* | G | 8.333333 |
| *Cryomorphaceae* | G | 8.166667 |
| unclassified_f__Clade_I | G | 8 |
| *Candidatus_Accumulibacter* | G | 7.25 |
| unclassified_f__Flavobacteriaceae | G | 6.416667 |
| unclassified_c__Bacteroidia | G | 6.083333 |
| norank_f__Sporolactobacillaceae | G | 5.666667 |
| Sva0996_marine_group | G | 5.5 |
| *Marinobacter* | G | 4.583333 |
| NS2b_marine_group | G | 4.583333 |
| hgcI_clade | G | 4.25 |
| OM43_clade | G | 4.083333 |
| *Fusobacterium* | G | 4 |
| *Marinoscillum* | G | 4 |
| norank_f__norank_o__norank_c__norank_p__WPS-2 | G | 4 |
| SUP05_cluster | G | 4 |

* There are 250 unique genera in the guts. Due to space constraints, only the top 25 unique genera with their relative abundances are displayed.

**SI Tab.4 List of cultured symbiotic bacteria of *H. venatoria***

| Isolate  Code | Accession No. | Closet match | GenBank No. | Similarity  (%) |
| --- | --- | --- | --- | --- |
| GB101 | OM267660 | *Bacillus wiedmannii* FSL W8-0169 ^T^ | LOBC01000053 | 99.24 |
| GB103 | ON231703 | *Bacillus cereus* ATCC 14579 ^T^ | AE016877 | 99.16 |
| GB201 | OM618000 | *Chryseobacterium vietnamense* GIMN1.005 ^T^ | HM212415 | 98.41 |
| GB211 | ON231679 | *Bacillus albus* N35-10-2 ^T^ | MAOE01000087 | 99.38 |
| GB212 | ON514243 | *Paenibacillus taichungensis* BCRC 17757 ^T^ | EU179327 | 98.9 |
| GB214 | ON514244 | *Pseudomonas brassicae* MAFF 212427 ^T^ | LC514379 | 99.72 |
| GB311 | ON514238 | *Bacillus velezensis*CR-502 ^T^ | AY603658 | 99.36 |
| GB312 | ON514239 | *Bacillus velezensis* CR-502 ^T^ | AY603658 | 99.5 |
| GB313 | ON514240 | *Bacillus altitudinis* 41KF2b ^T^ | ASJC01000029 | 99.04 |
| GK101 | OM267661 | *Bacillus albus* N35-10-2 ^T^ | MAOE01000087 | 99.04 |
| GK211 | ON548134 | *Paracoccus yeei* ATCC BAA-599 ^T^ | JHWH01000002 | 99.41 |
| GK212 | ON514241 | *Delftia tsuruhatensis* NBRC16741 ^T^ | BCTO01000107 | 99.24 |
| GK213 | ON514242 | *Bacillus albus* N35-10-2 ^T^ | MAOE01000087 | 98.43 |
| GL101 | ON459672 | *Bacillus velezensis*CR-502 ^T^ | AY603658 | 99.58 |
| GL102 | OL824862 | *Serratia marcescens* ATCC 13880 ^T^ | JMPQ01000005 | 98.61 |
| GL103 | OL824863 | *Serratia marcescens* ATCC 13880 ^T^ | JMPQ01000005 | 99.24 |
| GL104 | OL824864 | *Stenotrophomonas maltophilia* BII-R7 ^T^ | LT622838 | 99.28 |
| GL112 | OL824865 | *Bacillus wiedmannii* FSL W8-0169 ^T^ | LOBC01000053 | 98.72 |
| GL202 | OL824866 | *Pseudomonas oryzihabitans* NBRC 102199 ^T^ | BBIT01000012 | 99.3 |
| GL207 | OL824867 | *Bacillus siamensis* KCTC 13613 ^T^ | AJVF01000043 | 99.79 |
| GL211 | OL824868 | *Serratia marcescens* ATCC 13880 ^T^ | JMPQ01000005 | 99.38 |
| GL212 | OL824869 | *Prolinoborus fasciculus* CIP 103579 ^T^ | JN175353 | 99.02 |
| GL312 | OL824870 | *Priestia megaterium* NBRC 15308 ^T^ | JJMH01000057 | 99.25 |
| GL314 | OL824871 | *Serratia marcescens* ATCC 13880 ^T^ | JMPQ01000005 | 98.82 |
| GL315 | ON459673 | *Serratia marcescens* ATCC 13880 ^T^ | CP001686 | 98.18 |
| GM115 | ON514233 | *Delftiatsuruhatensis* NBRC 16741 ^T^ | BCTO01000107 | 99.23 |
| GM214 | ON514231 | *Bacillus albus* N35-10-2 ^T^ | MAOE01000087 | 99.1 |
| GM312 | ON514249 | *Rahnellaaceris* SAP-19 ^T^ | MN737201 | 99.03 |
| GM315 | ON514232 | *Bacillus paralicheniformis* KJ-16 ^T^ | KY694465 | 99.24 |
| GM322 | ON514248 | *Bacillus albus* N35-10-2 ^T^ | MAOE01000087 | 99.59 |
| GM331 | ON514235 | *Bacillus albus* N35-10-2 ^T^ | MAOE01000087 | 99.45 |
| GM332 | ON514236 | *Bacillus albus* N35-10-2 ^T^ | MAOE01000087 | 99.38 |
| GM333 | ON514237 | *Rahnellaaceris* SAP-19 ^T^ | MN737201 | 99.17 |
| GN201 | OM267658 | *Bacillus paramycoides* NH24A2 ^T^ | MAOI01000012 | 96.68 |

**SI Tab.4 Continued**

| Isolate Code | Accession No. | Closest match | GenBank No. | Similarity(%) |
| --- | --- | --- | --- | --- |
| GN202 | OM267659 | *Bacillus cereus* ATCC 14579^T^ | AE016877 | 99.24 |
| GN215 | ON514245 | *Paracoccusyeei*ATCC BAA-599^T^ | JHWH01000002 | 99.56 |
| GR213 | ON514234 | *Moraxella osloensis* CCUG 350^T^ | CP014234 | 98.67 |
| GR215 | ON514247 | *Delftia tsuruhatensis* NBRC 16741^T^ | BCTO01000107 | 99.51 |
| GR216 | ON514246 | *Delftia tsuruhatensis* NBRC 16741^T^ | BCTO01000107 | 99.24 |
| GT105 | OL824872 | *Lysinibacillus fusiformis* NBRC 15717^T^ | AB271743 | 99.04 |
| GT106 | OL824873 | *Bacillus paralicheniformis* KJ-16^T^ | KY694465 | 99.93 |
| GT107 | OL824874 | *Serratia marcescens* ATCC 13880^T^ | JMPQ01000005 | 99.04 |
| GT116 | OL824875 | *Serratia marcescens* ATCC 13880^T^ | JMPQ01000005 | 99.86 |
| GT208 | OL824876 | *Bacillus velezensis*CR-502^T^ | AY603658 | 99.78 |
| GT211 | OL824878 | *Bacillus albus*N35-10-2^T^ | MAOE01000087 | 99.11 |
| GT215 | OL824879 | *Serratia marcescens* ATCC 13880^T^ | JMPQ01000005 | 99.03 |
| GT311 | OL824880 | *Micrococcus cohnii* WS4601^T^ | FR832424 | 97.77 |
| GT312 | ON459674 | *Metabacillusidriensis* SMC 4352-2^T^ | AY904033 | 99.51 |
| GT313 | OL824881 | *Serratia marcescens* ATCC 13880^T^ | JMPQ01000005 | 99.44 |
| GT314 | OL824882 | *Serratia marcescens* ATCC 13880^T^ | JMPQ01000005 | 99.58 |
| GT318 | OL824883 | *Paraburkholderia insulsa* PNG-April^T^ | KF733462 | 99.65 |
| OB101 | ON231680 | *Bacillus wiedmannii* FSL W8-0169^T^ | LOBC01000053 | 99.52 |
| OB102 | ON231682 | *Bacillus albus* N35-10-2^T^ | MAOE01000087 | 99.04 |
| OB201 | OM618001 | *Chryseobacteriumindologenes*NBRC 14944^T^ | BAVL01000024 | 98.6 |
| OK201 | ON231681 | *Bacillus cereus* ATCC 14579^T^ | AE016877 | 99.52 |
| OK311 | ON231678 | *Bacillus albus* N35-10-2^T^ | MAOE01000087 | 98.84 |
| OL212 | OL824884 | *Planococcusglaciei*423^T^ | EU036220 | 98.71 |
| OL213 | OL824885 | *Serratia marcescens* ATCC 13880^T^ | JMPQ01000005 | 99.03 |
| OL217 | OL824886 | *Lysinibacillus fusiformis* NBRC 15717^T^ | AB271743 | 99.72 |
| OL302 | OL824887 | *Micrococcus endophyticus* YIM 56238^T^ | EU005372 | 98.93 |
| OL304 | OL824889 | *Paraburkholderiafungorum* NBRC 102489^T^ | BAYC01000104 | 99.86 |
| OL306 | OL824890 | *Brevundimonasvesicularis* NBRC 12165^T^ | BCWM01000033 | 99.85 |
| OL307 | OL824891 | *Serratia marcescens* ATCC 13880^T^ | JMPQ01000005 | 99.03 |
| OT104 | OL824892 | *Bacillus wiedmannii* FSL W8-0169^T^ | LOBC01000053 | 98.64 |
| OT202 | OL824893 | *Serratia marcescens* ATCC 13880^T^ | JMPQ01000005 | 98.61 |
| OT206 | OL824894 | *Pseudarcicellahirudinis*DSM 25647^T^ | jgi.1068048 | 98.43 |
| OT301 | OL824896 | *Brevundimonas faecalis* CS20.3^T^ | FR775448 | 99.09 |
| OT306 | OL824897 | *Pseudomonas oryzihabitans*NBRC 102199^T^ | BBIT01000012 | 98.57 |

**SI Tab.4 Continued**

| Isolate Code | Accession No. | Closest match | GenBank No. | Similarity  (%) |
| --- | --- | --- | --- | --- |
| PB202 | ON231684 | *Prolinoborus fasciculus* CIP 103579^T^ | ONZB01000010 | 99.64 |
| PB221 | ON231704 | *Bacillus paramycoides* NH24A2^T^ | MAOI01000012 | 98.61 |
| PK102 | ON231685 | *Exiguobacteriumenclense* NIO-1109^T^ | JF893462 | 99.15 |
| PL111 | OL824899 | *Bacillus albus* N35-10-2^T^ | MAOE01000087 | 98.9 |
| PL112 | ON459675 | *Chryseobacterium aquaticum* KCTC 12483^T^ | LLYZ01000003 | 98.8 |
| PL114 | OL824900 | *Agrobacterium deltaense* YIC 4121^T^ | MRDI01000025 | 99.21 |
| PL116 | OL824901 | *Microbacteriumproteolyticum* RZ36^T^ | KM359785 | 99.14 |
| PL117 | OL824902 | *Paraburkholderiainsulsa* PNG-April^T^ | KF733462 | 99.72 |
| PL118 | OL824903 | *Agrobacterium deltaense* YIC 4121^T^ | MRDI01000025 | 99.71 |
| PL119 | OL824904 | *Microbacteriumproteolyticum* RZ36^T^ | KM359785 | 99.14 |
| PL207 | ON459677 | *Chryseobacterium aquaticum* KCTC 12483^T^ | LLYZ01000003 | 98.73 |
| PL208 | ON459678 | *Bacillus cereus* NBRC 102489^T^ | BAYC01000104 | 100 |
| PL211 | OL824906 | *Bacillus zanthoxyli*1433^T^ | KX865140 | 99.58 |
| PL213 | OL824908 | *Bacillus wiedmannii*FSL W8-0169^T^ | LOBC01000053 | 99.04 |
| PL214 | OL824909 | *Brevundimonasvesicularis* NBRC 12165^T^ | BCWM01000033 | 99.09 |
| PL215 | OL824910 | *Paraburkholderiainsulsa*PNG-April^T^ | KF733462 | 99.36 |
| PL217 | ON459679 | *Chryseobacteriumaquaticum*KCTC 12483^T^ | LLYZ01000003 | 99.15 |
| PL218 | OL824911 | *Agrobacterium deltaense* YIC 4121^T^ | MRDI01000025 | 99.78 |
| PL219 | ON459680 | *Lysinibacillus fusiformis*. NBRC 15717^T^ | AB271743 | 99.24 |
| PL313 | ON459681 | *Chryseobacterium aquaticum* KCTC 12483^T^ | LLYZ01000003 | 98.66 |
| PL315 | OL824913 | *Serratia marcescens* ATCC 13880^T^ | JMPQ01000005 | 99.17 |
| PL316 | OL824914 | *Bacillus cereus ATCC* 14579^T^ | AE016877 | 99.59 |
| PL319 | ON459682 | *Agrobacterium deltaense* YIC 4121^T^ | MRDI01000025 | 98.99 |
| PM211 | OM617892 | *Staphylococcus saprophyticus* Bovis GTC 843^T^ | AB233327 | 99.45 |
| PR202 | ON231686 | *Bacillus albus* N35-10-2^T^ | NZ_MAOE01000 | 98.83 |
| PT112 | ON459683 | *Bacillus wiedmannii* FSL W8-0169^T^ | LOBC01000053 | 98.87 |
| PT114 | ON459684 | *Bacillus albus* N35-10-2^T^ | MAOE01000087 | 98.66 |
| PT118 | OL881265 | *Agrobacterium radiobacter* ATCC 19358^T^ | AJ389904 | 97.73 |
| PT211 | ON459685 | *Bacillus albus* N35-10-2^T^ | MAOE01000087 | 98.85 |
| PT212 | ON459686 | *Bacillus tequilensis* KCTC 13622^T^ | AYTO01000043 | 98.71 |
| PT213 | OL881266 | *Bacillus velezensis*CR-502^T^ | AY603658 | 100 |
| PT215 | OL881267 | *Bacillus manliponensis*BL4-6^T^ | FJ416490 | 98.78 |
| PT216 | OL824920 | *Agrobacterium deltaense* YIC 4121^T^ | MRDI01000025 | 97.82 |
| PT217 | OL881268 | *Agrobacterium fabrum* C58^T^ | AE007869 | 98.33 |

**SI Tab.4 Continued**

| Isolate Code | Accession No. | Closest match | GenBank No. | Similarity  (%) |
| --- | --- | --- | --- | --- |
| PT311 | ON459687 | *Chryseobacteriumaquaticu* KCTC 12483^T^ | LLYZ01000003 | 98.78 |
| PT312 | OL824921 | *Agrobacterium deltaense* YIC 4121^T^ | MRDI01000025 | 99.56 |
| PT315 | OL824922 | *Paraburkholderiafungorum* NBRC 102489^T^ | BAYC01000104 | 100 |
| PT316 | OL824923 | *Agrobacterium deltaense* YIC 4121^T^ | MRDI01000025 | 99.49 |
| PT317 | OL881269 | *Rhizobium arsenicireducens* KAs 5-22^T^ | JX173993 | 97.85 |
| SB302 | ON231702 | *Exiguobacteriumenclense* NIO-1109^T^ | JF893462 | 98.44 |
| SB311 | ON231687 | *Bacillus albus* N35-10-2^T^ | NZ_MAOE01000 | 98.69 |
| SL111 | OL824925 | *Serratia marcescens* ATCC 13880^T^ | JMPQ01000005 | 99.72 |
| SL112 | OL824926 | *Serratia marcescens* ATCC 13880^T^ | JMPQ01000005 | 99.03 |
| SL212 | OL824928 | *Bacillus paramycoides* NH24A2^T^ | MAOI01000012 | 98.78 |
| SL213 | OL824929 | *Serratia marcescens* ATCC 13880^T^ | JMPQ01000005 | 99.31 |
| SM211 | OM617894 | *Acinetobacter pittii* CIP 70.29^T^ | APQP01000001 | 98.9 |
| SN211 | OM617893 | *Lysinibacillus fusiformis* NBRC 15717^T^ | AB271743 | 98.97 |
| ST113 | OL824930 | *Bacillus wiedmannii* FSL W8-0169^T^ | LOBC01000053 | 99.18 |
| ST118 | OL824932 | *Serratia marcescens* ATCC 13880^T^ | JMPQ01000005 | 99.79 |
| ST211 | OL881271 | *Bacillus albus* N35-10-2^T^ | MAOE01000087 | 99.72 |
| ST212 | OL824933 | *Serratia marcescens*ATCC 13880^T^ | JMPQ01000005 | 99.79 |

Note: Strains from this study are denoted as scientific names and strain numbers. The first character of the strain number represented the tissue (G-gut, P-venom gland, O-ovary, S-silk gland), the second character represented the isolation medium (B- BHI, K-CASO AGAR, L-LB, M-MRS, N-C/10 MEDIUM R- R AGAR, T-TB2).

**SI Tab.5 Taxonomy status of the bacteria isolated from different tissues**

| **Phyla** | **Genera** | **Numbers of isolates in different tissues** | | | | |
| --- | --- | --- | --- | --- | --- | --- |
|  |  | Gut | Ovary | Venom gland | Silk Gland | Total |
| Proteobacteria | *Serratia* | 11 | 3 | 1 | 5 | 20 |
|  | *Agrobacterium* | 0 | 0 | 9 | 0 | 9 |
|  | *Pseudomonas* | 2 | 1 | 0 | 0 | 3 |
|  | *Paraburkholderia* | 1 | 1 | 3 | 0 | 5 |
|  | *Delftia* | 4 | 0 | 0 | 0 | 4 |
|  | *Brevundimonas* | 0 | 2 | 1 | 0 | 3 |
|  | *Paracoccus* | 2 | 0 | 0 | 0 | 2 |
|  | *Rhizobium* | 0 | 0 | 1 | 0 | 1 |
|  | *Prolinoborus* | 1 | 0 | 1 | 0 | 2 |
|  | *Rahnella* | 2 | 0 | 0 | 0 | 2 |
|  | *Acinetobacter* | 0 | 0 | 0 | 1 | 1 |
|  | *Moraxella* | 1 | 0 | 0 | 0 | 1 |
|  | *Stenotrophomonas* | 1 | 0 | 0 | 0 | 1 |
| Firmicutes | *Bacillus* | 20 | 5 | 13 | 4 | 42 |
|  | *Lysinibacillus* | 1 | 1 | 1 | 1 | 4 |
|  | *Exiguobacterium* | 0 | 0 | 1 | 1 | 2 |
|  | *Priestia* | 1 | 0 | 0 | 0 | 1 |
|  | *Metabacillus* | 1 | 0 | 0 | 0 | 1 |
|  | *Planococcus* | 0 | 1 | 0 | 0 | 1 |
|  | *Paenibacillus* | 1 | 0 | 0 | 0 | 1 |
|  | *Staphylococcus* | 0 | 0 | 1 | 0 | 1 |
| Bacteroidota | *Chryseobacterium* | 1 | 1 | 5 | 0 | 7 |
|  | *Pseudarcicella* | 0 | 1 | 0 | 0 | 1 |
| Actinobacteriota | *Microbacterium* | 0 | 0 | 2 | 0 | 2 |
|  | *Micrococcus* | 1 | 1 | 0 | 0 | 2 |
| Total |  | 51 | 17 | 39 | 12 | 119 |

| Taxonomy | G_H | G_C | OC_H | OC_C | VG_H | VG_C | SG_H | SG_C |
| --- | --- | --- | --- | --- | --- | --- | --- | --- |
| *Acinetobacter* | 13.1564 | 0 | 5.4376 | 0 | 6.1572 | 0 | 0.8167 | 8.333333333 |
| *Agrobacterium* | 0 | 0 | 0 | 0 | 0 | 23.07692308 | 0 | 0 |
| *Bacillus* | 3.7859 | 39.21568627 | 90.2883 | 29.41176471 | 77.4439 | 33.33333333 | 81.9684 | 33.33333333 |
| *Brevundimonas* | 0 | 0 | 0 | 11.76470588 | 0 | 2.564102564 | 0 | 0 |
| *Candidatus_Cardinium* | 0.0016 | 0 | 0.0016 | 0 | 0.0063 | 0 | 0.1185 | 0 |
| *Candidatus_Rhabdochlamydia* | 0.0024 | 0 | 0.0071 | 0 | 0.0032 | 0 | 0.0103 | 0 |
| *Chryseobacterium* | 0.0253 | 1.960784314 | 0.0063 | 5.882352941 | 0.1556 | 12.82051282 | 0.015 | 0 |
| *Cloacibacterium* | 0.8523 | 0 | 0.0166 | 0 | 0.019 | 0 | 0.0142 | 0 |
| *Delftia* | 3.9147 | 7.843137255 | 0.1872 | 0 | 0.2891 | 0 | 0.3847 | 0 |
| *Exiguobacterium* | 0 | 0 | 0 | 0 | 0 | 2.564102564 | 0 | 8.333333333 |
| *Flavobacterium* | 0.3547 | 0 | 0.113 | 0 | 0.0853 | 0 | 0.1177 | 0 |
| *Lactobacillus* | 0.6043 | 0 | 0.2646 | 0 | 0.1161 | 0 | 0.8981 | 0 |
| *Lysinibacillus* | 0 | 1.960784314 | 0 | 5.882352941 | 0 | 2.564102564 | 0 | 8.333333333 |
| *Metabacillus* | 0 | 1.960784314 | 0 | 0 | 0 | 0 | 0 | 0 |
| *Microbacterium* | 0 | 0 | 0 | 0 | 0 | 5.128205128 | 0 | 0 |
| *Micrococcus* | 0 | 1.960784314 | 0 | 5.882352941 | 0 | 0 | 0 | 0 |
| *Moraxella* | 0 | 1.960784314 | 0 | 0 | 0 | 0 | 0 | 0 |

**SI Tab.6 List of relative abundance of top 30genera of HTS and Culturomics of *H. venatoria***

**SI Tab.6 Continued**

| Taxonomy | G_H | G_C | OC_H | OC_C | VG_H | VG_C | SG_H | SG_C |
| --- | --- | --- | --- | --- | --- | --- | --- | --- |
| *Morganella* | 0.2512 | 0 | 0.015 | 0 | 0.0134 | 0 | 0.1343 | 0 |
| *Novosphingobium* | 0.4558 | 0 | 0.0063 | 0 | 0.0182 | 0 | 0.0024 | 0 |
| *Ochrobactrum* | 0.0134 | 0 | 0.113 | 0 | 0.549 | 0 | 0.0284 | 0 |
| Others | 20.7875 | 0 | 1.2441 | 0 | 10.0585 | 0 | 8.0782 | 0 |
| *Paenibacillus* | 0 | 1.960784314 | 0 | 0 | 0 | 0 | 0 | 0 |
| *Parabacteroides* | 0.6643 | 0 | 0.1019 | 0 | 0.0458 | 0 | 2.3531 | 0 |
| *Paraburkholderia* | 0 | 1.960784314 | 0 | 5.882352941 | 0 | 7.692307692 | 0 | 0 |
| *Paracoccus* | 0 | 3.921568627 | 0 | 0 | 0 | 0 | 0 | 0 |
| *Phyllobacterium* | 6.8207 | 0 | 0.1619 | 0 | 0.2243 | 0 | 0.2773 | 0 |
| *Planococcus* | 0 | 0 | 0 | 5.882352941 | 0 | 0 | 0 | 0 |
| *Polaribacter* | 0.2156 | 0 | 0.0008 | 0 | 0.0008 | 0 | 0 | 0 |
| *Priestia* | 0 | 1.960784314 | 0 | 0 | 0 | 0 | 0 | 0 |
| *Prolinoborus* | 0 | 1.960784314 | 0 | 0 | 0 | 2.564102564 | 0 | 0 |
| *Pseudarcicella* | 0 | 0 | 0 | 5.882352941 | 0 | 0 | 0 | 0 |
| *Pseudomonas* | 0.4329 | 3.921568627 | 0.4739 | 5.882352941 | 1.248 | 0 | 0.2227 | 0 |
| *Rahnella* | 0 | 3.921568627 | 0 | 0 | 0 | 0 | 0 | 0 |
| *Ralstonia* | 4.154 | 0 | 0.1848 | 0 | 0.3847 | 0 | 0.5671 | 0 |

**SI Tab.6 Continued**

| Taxonomy | G_H | G_C | OC_H | OC_C | VG_H | VG_C | SG_H | SG_C |
| --- | --- | --- | --- | --- | --- | --- | --- | --- |
| *Rhizobium* | 0 | 0 | 0 | 0 | 0 | 2.564102564 | 0 | 0 |
| *Rickettsia* | 0.1572 | 0 | 0.0174 | 0 | 0.0118 | 0 | 0.0142 | 0 |
| *Rickettsiella* | 0.0727 | 0 | 0.0395 | 0 | 0.034 | 0 | 0.0474 | 0 |
| *Serratia* | 0 | 21.56862745 | 0 | 17.64705882 | 0 | 2.564102564 | 0 | 41.66666667 |
| *Simplicispira* | 0.3799 | 0 | 0.019 | 0 | 0.0095 | 0 | 0.0158 | 0 |
| *Sphingobacterium* | 0.0016 | 0 | 0.0126 | 0 | 0.0253 | 0 | 0 | 0 |
| *Sphingomonas* | 5.7528 | 0 | 0.0782 | 0 | 0.2362 | 0 | 0.3705 | 0 |
| *Staphylococcus* | 0 | 0 | 0 | 0 | 0 | 2.564102564 | 0 | 0 |
| *Stenotrophomonas* | 26.7701 | 1.960784314 | 0.7101 | 0 | 2.0213 | 0 | 2.8302 | 0 |
| *Thauera* | 0.6469 | 0 | 0.2994 | 0 | 0.2441 | 0 | 0.2583 | 0 |
| *Tsukamurella* | 0.0237 | 0 | 0.0174 | 0 | 0.0395 | 0 | 0.0158 | 0 |
| *unidentified_Alphaproteobacteria* | 0.8942 | 0 | 0.0016 | 0 | 0.0024 | 0 | 0.0055 | 0 |
| *unidentified_Cyanobacteria* | 6.1082 | 0 | 0.06 | 0 | 0.4637 | 0 | 0.2101 | 0 |
| *unidentified_Rhizobiaceae* | 0.5687 | 0 | 0.0032 | 0 | 0.0679 | 0 | 0.1983 | 0 |
| *unidentified_Rhodocyclaceae* | 2.1122 | 0 | 0.0174 | 0 | 0.0118 | 0 | 0.0047 | 0 |
| *Wolbachia* | 0.019 | 0 | 0.1003 | 0 | 0.0142 | 0 | 0.0221 | 0 |
